# Mechanism Development and Evaluation of the Gas-Phase Photooxidation of Guaiacol: Insights from a Chamber Study

**DOI:** 10.1021/acsestair.5c00420

**Published:** 2026-03-16

**Authors:** Rhianna L. Evans, Rubén Soler, Teresa Vera, Mila Ródenas, Esther Borrás, Tatiana Gómez, Daniel J. Bryant, Alfred W. Mayhew, David R. Shaw, Simon P. O’Meara, Amalia Muñoz, Jacqueline F. Hamilton, Andrew R. Rickard

**Affiliations:** † Wolfson Atmospheric Chemistry Laboratories, Department of Chemistry, 8748 University of York, York YO10 5DD, United Kingdom; ‡ EUPHORE Laboratories, Fundación Centro de Estudios Ambientales del Mediterráneo (CEAM), 46980 Paterna, Spain; § Department of Atmospheric Sciences, 7060 University of Utah, Salt Lake City, Utah 84112, United States; ∥ National Centre for Atmospheric Science, University of York, York YO10 5DD, United Kingdom; ⊥ Department of Environment and Geography, University of York, York YO10 5DD, United Kingdom; # National Centre for Atmospheric Science, 5292 University of Manchester, Manchester M13 9PL, United Kingdom

**Keywords:** biomass burning, guaiacol, phenolic compounds, atmospheric oxidation, secondary organic aerosol, master chemical mechanism

## Abstract

Guaiacol (2-methoxyphenol) is an oxygenated aromatic volatile organic compound (VOC) emitted from biomass burning during lignin pyrolysis with significant potential to impact air quality through tropospheric ozone (O_3_) production and secondary organic aerosol (SOA) formation. In this work, a near-explicit mechanism for the photooxidation of guaiacol was developed for the inclusion into the Master Chemical Mechanism (MCM) framework, to improve the mechanistic understanding of the chemistry occurring in biomass burning plumes. The mechanism is evaluated using an outdoor smog chamber experiment conducted at the EUropean PHOtoREactor in Valencia, Spain. Model-measurement comparisons replicate chamber experiment profiles of first- and second-generation products reasonably well. Simulations also indicated that the hydroxyl radical (OH) production was sensitive to the ratio of ring-retained and ring-opened products from first- and second-generation products, with increased formation from the ring opening pathways. However, despite optimization of the mechanism, several deficiencies remained in the model, which require further constraining: (1) overestimation of the O_3_ concentration, (2) underestimation of guaiacol loss from reaction with OH, and (3) overestimation of the rate of conversion from nitric oxide (NO) to nitrogen dioxide (NO_2_). An investigation into the composition of particle-phase guaiacol products was also evaluated using the CHemistry with Aerosol Microphysics in Python (PyCHAM) chamber box model, which predicted a significant contribution of nitroaromatics to early-stage guaiacol SOA formation with potentially important implications for air quality and climate from biomass burning.

## Graphical abstract

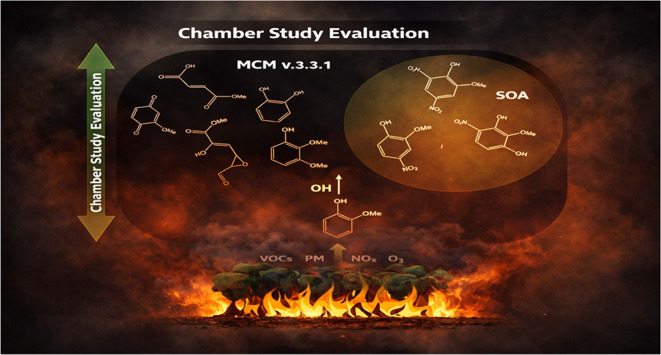

## 1 Introduction

Biomass burning is the second largest global source of nonmethane organic compounds (500 Tg yr^–1^) to the atmosphere after biogenic emissions (1000 Tg yr^–1^)^1^ and the dominant source of primary organic aerosol (POA) and black carbon (BC).^2^ Emissions of biomass burning volatile organic compounds (BBVOCs) can undergo photooxidation in the atmosphere to produce gaseous and particle-phase species such as secondary organic aerosol (SOA) and ozone (O_3_) in the presence of nitrogen oxides (NO_*x*_ = NO + NO_2_). These emissions and their products can impact on climate by affecting the radiation budget, plant productivity through stomatal uptake of O_3_, and human health.^3–5^ As a result of climate change and anthropogenic activity, the intensity, frequency, and severity of biomass burning are increasing.^6^ However, despite the importance of biomass burning for atmospheric composition and the resulting impacts, the atmospheric chemistry of biomass burning emissions remains poorly understood.

Monoterpenes and oxygenated aromatics (i.e., furans and phenols) are considered the main sources of reactivity from biomass burning^7–9^ and have been reported as the dominant SOA precursors.^10^ An important class of oxygenated aromatics from biomass burning emissions are methoxyphenols, which form during the pyrolysis of lignin^11–13^ and can vary depending on the plant species undergoing combustion.^14^ For example, in softwoods the methoxyphenols are primarily guaiacyl units, while hardwoods contain equal amounts of guaiacyl and syringyl units,^13^ which overall can result in a significant emission of guaiacol from biomass burning (approximately 172–276 mg kg^–1^).^15^ Guaiacol is highly reactive toward OH (*k*_OH_ = 5.40–7.53 × 10^–11^ cm^3^ molecule^–1^ s^–1^)^16,17^ and the reported SOA yields can also be significant (0.003–0.87) depending on the oxidative conditions,^18–20^ which could have significant implications for air quality.

Despite the atmospheric significance of guaiacol, to date the majority of theoretical and laboratory studies on oxygenated aromatics^7,20–26^ have yet to propose a detailed chemical mechanism for the atmospheric photooxidation of guaiacol for inclusion into chemical modeling frameworks. These frameworks such as the Master Chemical Mechanism (MCM, https://mcm.york.ac.uk/MCM) can enable an improved understanding surrounding the impact of biomass burning on air quality and climate in both present and future scenarios. Previous studies investigating the reaction of guaiacol with OH have largely performed theoretical calculations to understand the energetically favorable H-abstraction and OH addition pathways,^27–30^ and in some experimental studies basic reaction schemes were provided based on mass spectrometry measurements.^18,20^ From the H-abstraction pathway, Lauraguais et al.^18^ reported that nitroguaiacols were the dominant gas-phase products under high NO_*x*_ conditions, with yields of 16% and the high potential of these compounds to partition to the aerosol phase where they are known contributors to brown carbon (BrC).^31–37^ In computational studies of guaiacol photooxidation^27–30^ and in experiments performed by Yee et al.,^20^ under low NO_*x*_ conditions, OH addition products such as methoxybenzoquinone, methoxybenzene-1,4-diols, unsaturated dicarbonyls, and epoxides were also observed. In computational studies, intermediates deriving from the H-abstraction of the methoxy group^29^ and OH addition on the ring at the ortho and ipso positions to the methoxy group^27,30^ were considered the most energetically favorable pathways, also yielding nitroguaiacols under high NO_*x*_ conditions.^18,27,30^ Furthermore, An et al.^27^ suggest H-abstraction reaction pathways were dominant over OH addition reactions, which may have implications for the SOA toxicity due to the significant formation of ring-retained products from this pathway, which had higher oxidative potentials than ring-opened products in guaiacol SOA as measured by dithiothreitol (DTT) assays.^38^

This study proposes a newly developed mechanism for the photooxidation of guaiacol using the latest literature and MCM development protocols. The performance of the new guaiacol mechanism was evaluated against a chamber study performed at the EUropean PHOtoREactor (EUPHORE) in May 2023. Shortcomings in the proposed mechanism are identified and discussed.

## 2 Methods

### 2.1 Mechanism Development

The guaiacol mechanism was constructed using the latest literature and MCM mechanism development protocols,^39–41^ coupled to the updated Structure–Activity Relationship parametrizations for the OH oxidation of aliphatic species, aromatics, and the degradation of peroxy radicals (RO_2_)^42–44^ and merged with the current MCM v3.3.1 subset.

#### 2.1.1 Derivation of the *k*_OH_ Rate Coefficient

Generally, the MCM protocols use kinetic data that have been extensively reviewed or, if available, more recent observations from the literature.^45–47^ However, where neither recommendation nor experimental/theoretical data exist, the rate coefficients are approximated using Structure–Activity Relationships (SARs), which apply a set of generic partial rate coefficient estimations that are modified depending on the carbon structure and functional groups present, or the rate coefficient of a close analogue can be applied. To date, there are few experimental rate coefficients for the reaction of guaiacol with the OH radical with values of *k*_OH_ ranging between 5.40 and 7.53 × 10^–11^ cm^3^ molecule^–1^ s^–1^ at 294–295 K.^16,17^ However, in this approach, we also calculate the *k*_OH_ from the SAR protocol described in Jenkin et al.^42^ to be 6.99 × 10^–11^ cm^3^ molecule^–1^ s^–1^ at 298 K, which is in good agreement within this experimental range.^16,17^ An estimated rate for the reaction of guaiacol with NO_3_ was also determined using the GECKO-A SAR calculator^48,49^ and deemed relatively unimportant in the daytime due to NO_3_ photolysis. However, it is important to note that experimental studies have reported rate coefficients that differ by 1 order of magnitude (*k*_NO_3__ = 3.2 × 10^–12^–3.1 × 10^–11^ cm^3^ molecule^–1^ s^–1^ at 294 K)^25,50^ and future work may be needed to evaluate the potential importance of daytime NO_3_ chemistry.^51,52^

#### 2.1.2 Determination of the Initial Branching Ratios to First-Generation Products

The partial rate coefficients determined in Section 2.1.1 for each OH reaction site were used to derive the initial branching ratios to the first-generation guaiacol products as in the aromatic SAR protocol.^42^ In the SAR prediction, the initial branching ratio for the H-abstraction of the OH group, which typically yields nitrophenols,^18,27,30^ was low (3.7%) and would typically be discounted by the MCM protocol for pathways with less than 5% yield.^39^ However, Lauraguais et al.^18^ observed a nitroguaiacol yield of 16%, which is used instead, assuming the resulting phenoxy radical exclusively reacts with NO_2_ to produce nitroguaiacol. This is a similar approach used for other phenolic compounds in the MCM; however, this assumption ignores the other possible products that may form via this pathway such as quinones.^53^ Previous studies have also observed the formation of catechol and catechol oxidation products (e.g., 4-nitrocatechol)^18,20,54^ from guaiacol oxidation, which is proposed to occur from OH addition ipso to the methoxy (–OMe) group^54^ and has a branching ratio of 6% at this position. The branching ratio for the H-abstraction of the OMe group was low (<5%), and according to protocol rules, was removed and its partial reactivity redistributed among the other channels. Furthermore, theoretical calculations predominantly observed H-abstraction of the OMe group forms nitroguaiacol^30^ that is already accounted for and therefore enables the scheme to be simplified. To calculate the branching ratios for other pathways forming the bicyclic peroxy radical, epoxide, and OH addition products, the site with the highest partial rate coefficient was chosen, which in this case was ortho OH addition, yielding the results outlined in Scheme S1. However, alternatively using the para OH addition partial rate coefficient would produce a similar branching distribution (±10%) to Scheme S1.

#### 2.1.3 Reactions of Peroxy Radicals (RO_2_)

RO_2_ can undergo bimolecular reactions with species such as NO, NO_2_, HO_2_, and NO_3_, as well as RO_2_–RO_2_ cross and self-reactions.^43^ The rates of these reactions ultimately depend on the ambient conditions and the RO_2_ structure (i.e., primary, secondary, tertiary). Generally, the calculation of RO_2_ bimolecular rate coefficients is already built into the MCM. However, for RO_2_ cross and self-reactions, the equations to determine the rate coefficient and branching ratios in the Jenkin et al.^43^ parametrization are implemented. The degradation pathways of guaiacol with OH are shown in Scheme 1, which leads to a range of ring-opened and ring-closed products. However, future work should aim to incorporate a parametrization for potential intramolecular RO_2_ chemistry for aromatic compounds, which may be an important potential source of radicals and SOA, even under relatively high NO conditions but is not currently accounted for in the MCM protocol.^55^

**Scheme 1:**
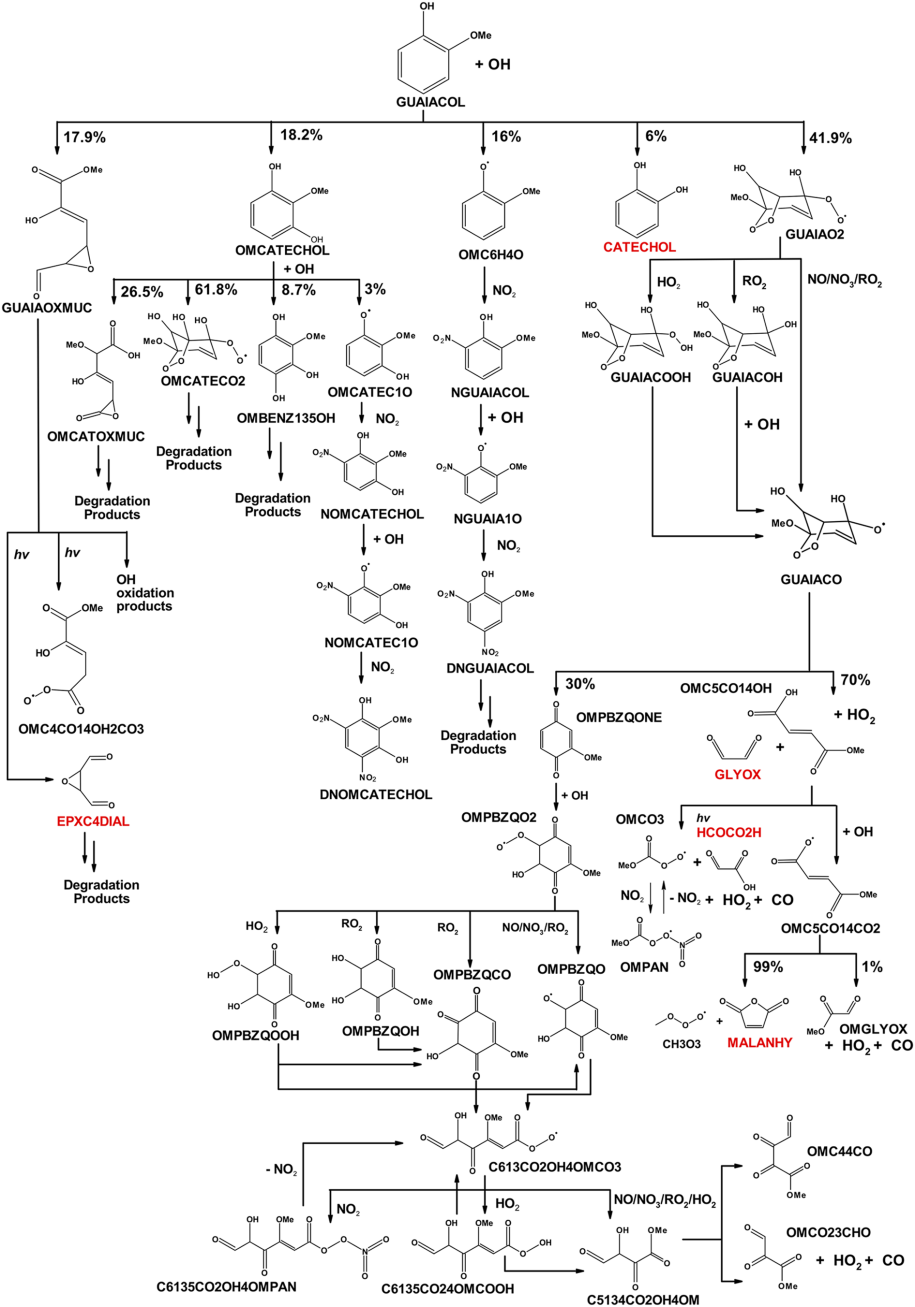
Guaiacol Photooxidation Mechanism Derived Using SAR Calculations until the Fourth Generation^a^

a Compound names shown in red are species, which have represented chemistry in the current MCM framework whilst those in black are added in this work.

#### 2.1.4 Reactions of Guaiacol Oxidation Products

Many of the first-generation products can undergo further chemistry through reactions with OH, NO_3_, or O_3_ or photolysis. For example, the formation of nitroguaiacol is anticipated to further react with OH (and NO_3_) radicals via H-abstraction of the hydroxyl group and in the presence of NO_*x*_ forms a dinitroguaiacol compound.^54^ Dinitroguaiacol is also predicted to undergo further reactions following a chemical degradation scheme similar to the existing dinitroaromatic schemes in the MCM.

While epoxides are a significant product in the oxidation of isoprene, with reported yields of up to 80%,^56,57^ in aromatic systems they generally form in lower yields (∼10%).^58,59^ However, epoxides can have important implications for toxicity^60^ and in the gas phase can react further with OH and degrade by photolysis (Scheme 1).

OH rate coefficients and subsequent products were calculated for the OH addition hydroxyarene products, such as OMCATECHOL and OMBENZ135OH using the Jenkin et al.^42^ SAR. The added chemistry subset for the subsequent RO_2_ degradation^43^ of the OH addition product OMCATECHOL is shown in Scheme S2 until the fourth generation; however, further efforts are needed to evaluate the impact of chemistry beyond this generation.

In total, the newly developed OH + guaiacol mechanism (Scheme 1) contains 414 reactions and 134 species up to the fourth generation, which is a similar size to that of benzene in the MCM. The optimized guaiacol scheme developed in this work is available in the Supporting Information.

### 2.2 Mechanism Evaluation

#### 2.2.1 Chamber Experiments

Chamber experiments were performed at the EUropean PHOtoREactor (EUPHORE), in Valencia, Spain, during May 2023 for a range of biomass burning precursors; however, the data presented in this study was collected from a gas-phase guaiacol photooxidation experiment performed on 15 May 2023. The EUPHORE facility has previously been described in detail.^61–63^ In summary, it comprises two 200 m^3^ Teflon outdoor chambers, where each chamber has two large internal fans for mixing and a retractable housing to enable chamber irradiation by sunlight or remain closed for dark experiments. Several important gas-phase species were measured for the assessment of the mechanism performance such as NO and NO_2_ using chemiluminescence and cavity-attenuated phase shift, respectively (models T2000UP and T500U, Teledyne, USA). However, Fourier Transform Infrared Spectroscopy (FTIR) measured guaiacol, O_3_, formaldehyde (HCHO), nitric acid (HNO_3_), nitrous acid (HONO), and glyoxal among other compounds. Proton Transfer Time-of-Flight Mass Spectrometry (PTR-ToF-MS, Ionicon Analytik, Austria) and Iodide-adduct High-Resolution Time-of-Flight Chemical Ionization Mass Spectrometry (Iodide HR-ToF-CIMS, Aerodyne Research Inc., USA) were used to measure guaiacol oxidation products. For the PTR-ToF-MS measurements, heated and inert sampling lines were used (stainless steel followed by heated PEEK, 80 °C) with an inlet length of 1.5 m at a flow rate of 89 mL min^–1^.

For the HR-API-TOF-CIMS measurements, sampling was performed through a 1.5 m long, 1/4″ diameter Teflon inlet operated at a flow rate of 2 L min^–1^. Mass spectra from Iodide HR-ToF-CIMS (I-CIMS) and PTR-ToF-MS were analyzed in PTRMS_Viewer 3.4 (Ionicon Analytik, Austria) and Tofware v4.0.2 in Igor Pro 8, respectively. The I-CIMS data was normalized using the sum of the reagent ions (I^–^ + IH_2_O^–^) at each timestamp. The PTR-MS data was normalized against the proton signal (H_3_O^+^, *m*/*z* = 19) at each time step. A Scanning Mobility Particle Sizer (SMPS, Model 3082 and 3775, TSI, USA) scanning across the range 1–1000 at a flow rate of 0.3 L min^–1^ was used to measure particle mass concentrations and monitor guaiacol SOA formation in real time. The initial NO_2_ was chemically produced inside the chamber from the reaction of NO and aqueous O_3_. A pen-ray lamp with a flow of 99.9% purity oxygen at a pressure of 2 bar was used to generate O_3_, and NO was introduced to the chamber via a gas cylinder standard (5000 ppm) for this reaction. The dilution of the chamber was measured via FTIR using 6 mL of sulfur hexafluoride (SF_6_) as a tracer.

Approximately 500 ppb (441 ppb after stabilization) guaiacol (>99%, Sigma-Aldrich) and ca. 100 ppb total NO_*x*_ (36 ppb NO + 43 ppb NO_2_) were added to the chamber prior to opening, resulting in an approximate VOC:NO_*x*_ ratio of 5. Previously, it was reported that a VOC:NO_*x*_ < 8 is indicative of a VOC-limited O_3_ production regime,^64^ which is typically observed from fresh emissions.^65,66^ Therefore, this experiment could represent the guaiacol chemistry within a fresh biomass burning plume. The experiment was performed with the roof open for approximately 5 h in total, under dry conditions (RH < 1%), unseeded and at an average temperature of 29 °C. OH was generated through the photolysis of ∼40 ppb nitrous acid (HONO) inside the chamber.

The initial conditions used in this study are similar to those used in previous campaigns at EUPHORE to understand the photooxidation of aromatic VOCs^58,67^ and the reported VOC:NO_*x*_ from FIREX-AQ campaigns.^7^ However, our initial guaiacol concentration is significantly higher than that previously reported in the FIREX-AQ campaign during controlled burn experiments (40 ppb).^68^ Higher precursor concentrations were used in this study for the temporal observation of lower-concentration oxidation products used in the evaluation/optimization of the mechanism, such as glyoxal measurements by FTIR. Future experimental design using I-CIMS measurements may be more suited to lower precursor concentrations as reductions in the I-CIMS reagent ion counts, owing to the high VOC loading, were observed in the later stages of the experiment, meaning only data from the initial period was used in the evaluation of the mechanism. Furthermore, the particle growth largely occurs within the first 90 min (Figure S1) despite further additions of HONO (10:01am (UTC)) and guaiacol (11:17am (UTC)); therefore, we evaluate the mechanism in this initial growth period. Future work could explore this observation in particle growth in greater detail; however, it is beyond the scope of the current study. At the end of the experiment, a filter (Whatmann Quartz) was sampled at a flow rate of 11.5 L min^–1^ for 1 h for detailed offline chemical composition analysis, extracted with methanol (Optima LC/MS grade, Thermo Fisher) and analyzed via offline Ultra-High-Performance Liquid Chromatography coupled to High-Resolution Mass Spectrometry in negative ionization mode, and a detailed methodology can be found in the Supporting Information.

#### 2.2.2 Chamber Box Modeling of Gaseous and Particulate Phases

Gas-phase chamber box models of the guaiacol chamber experiment were run using the AtChem2 (v1.2.2) open source zero-dimensional box model^69^ and compared to the experimental data obtained from the EUPHORE guaiacol experiment. The models were constrained with measurements of RH, temperature, HONO, and the measured photolysis rate of NO_2_ (*j*_NO_2__). Concentrations of NO, NO_2_, O_3_, and guaiacol among other products were initialized but not constrained to enable their simulated temporal profiles to be compared to the experimental data. In some cases, the mechanism was adjusted to fit the observed data, in the case of over- or underprediction. Photolysis rates were calculated by MCM parametrization and scaled using the measured *j*_NO_2__.^41^ An auxiliary mechanism of the chemistry that can occur on the chamber walls from wall deposition and photolysis^70^ was also inputted using reference chamber experiments and the parameters described in previous studies.^71,72^

The zero-dimensional CHemistry with Aerosol Microphysics in Python (PyCHAM) (v5.6.0) computer box model^73^ was coupled with the guaiacol mechanism to investigate whether the observed gas-phase oxidation products of guaiacol were directly affected by partitioning to particles and/or the wall. The MCM v3.3.1 for inorganics was extended with the guaiacol scheme and the EUPHORE auxiliary mechanism described above.

To investigate the effect of particle and wall sinks on gas-phase concentrations with as much certainty as possible, PyCHAM model simulations were constrained by using several of the observed gas-phase temporal profiles: NO, NO_2_, O_3_, HONO, and HCHO. Nucleation parameters were manually tuned to give reasonable agreement between simulated and observed particle number concentration in the first 9 min of the experiment. Gas-wall partitioning was estimated through the method of Huang et al.,^74^ coupled to the EUPHORE chamber dimensions. Uncertainty in the effective absorbing mass of wall (*C*_w_) and the eddy diffusivity coefficient (*k*_e_)^74^ was explored by varying both by 2 orders of magnitude. *C*_w_ was varied between 1 × 10^–4^ g m^–3^ and 1 × 10^–2^ g m^–3^ according to suggested parameters for Teflon walls.^75,76^ However, *k*_e_ was varied between 4 × 10^–3^ s^–1^ (a conservative minimum based on the mixing rate of the EUPHORE chamber fans^61^ and assuming *k*_e_ = mixing time^–1^) and 4 × 10^–1^ s^–1^ (close to the value given by definition by Huang et al.^74^).

As the objective of the PyCHAM modeling was to simulate the effect of realistic heterogeneous sinks on gas-phase concentrations, the simulated total particle mass concentration was evaluated against observations. Model-observation comparisons for particle mass concentration across the wall partitioning uncertainty range are given in Figure S2. In the initial PyCHAM setup simulations of newly nucleated particles, they did not grow at observed rates. However, by allowing guaiacol oxidation to yield an extremely low volatility product (ELVOC, potentially resulting from autoxidation^77^ and detailed in the SI), observed growth of the newly nucleated particles could be well reproduced in the PyCHAM simulation (Figure S2), thereby allowing further particle growth through condensation of higher volatility guaiacol oxidation products. Vapor pressures of components were estimated using the method reported by Nannoolal et al.,^78^ which has previously returned relatively good agreement with observations.^79^ It was found that a combination of 1 × 10^–3^ g m^–3^ for *C*_w_ and 4 × 10^–1^ s^–1^ for *k*_e_ gave the closest agreement with observation (Figure S2); therefore, this setup was used for further comparisons of PyCHAM simulated concentrations and observed gas-phase components such as guaiacol shown in Figure S3. Although the testing for uncertainty across the box modeling parameter space is nonexhaustive, through constraining with observations of key inorganics, reproducing the observed particulate matter mass concentration, and using wall parameters consistent with literature reports, an optimal PyCHAM model setup (within the available constraints) for simulating the phenomenology of gas-phase concentrations is achieved.

## 3 Results and Discussion

### 3.1 Model-Measurement Comparison of the Observed Chemistry

#### 3.1.1 Evaluation of Precursor Loss and OH Production

The newly developed mechanism was evaluated by comparing the chamber experimental concentrations to the simulated concentrations from the AtChem2 chamber box models. The temporal evolution of key inorganic and organic oxidation markers, such as NO, NO_2_, O_3_, HCHO, HNO_3_, and glyoxal, as well as the guaiacol decay were used for initial comparison.

Using the initial mechanism in Scheme 1, which excludes the additional chemistry of the OH addition product OMCATECHOL RO_2_ (OMCATECO2, Scheme S2), the modeled decay of guaiacol is too slow leading to an overprediction of approximately 150 ppb compared to the FTIR measurement (Figure 1), suggesting the system reactivity is underpredicted. Reactivity is defined here as the OH loss of guaiacol, which is the dominant sink of guaiacol under the experimental conditions in this study. However, after inclusion of the OMCATECHOL degradation mechanism (Scheme S2), the modeled guaiacol concentration is improved, especially in the early stages of the guaiacol decay, compared to the observations but remains overpredicted by approximately 60 ppb at the end of the experiment (Figure 1). This indicates the radical propagation through OMCATECO2 could be an important ring opening degradation pathway within the early stages of the mechanism for increasing the reactivity and hence increasing guaiacol loss. The model simulations presented in Figure 1 use the SAR rate coefficient of *k*_guaiacol+OH_, from Section 2.1.1; however, increasing the rate coefficient to that derived in previous experiments resulted in minimal impact on guaiacol loss, indicating the missing reactivity could occur from underproduction of OH from reactions of guaiacol oxidation products in the chemical mechanism.^16^

**Figure 1:**
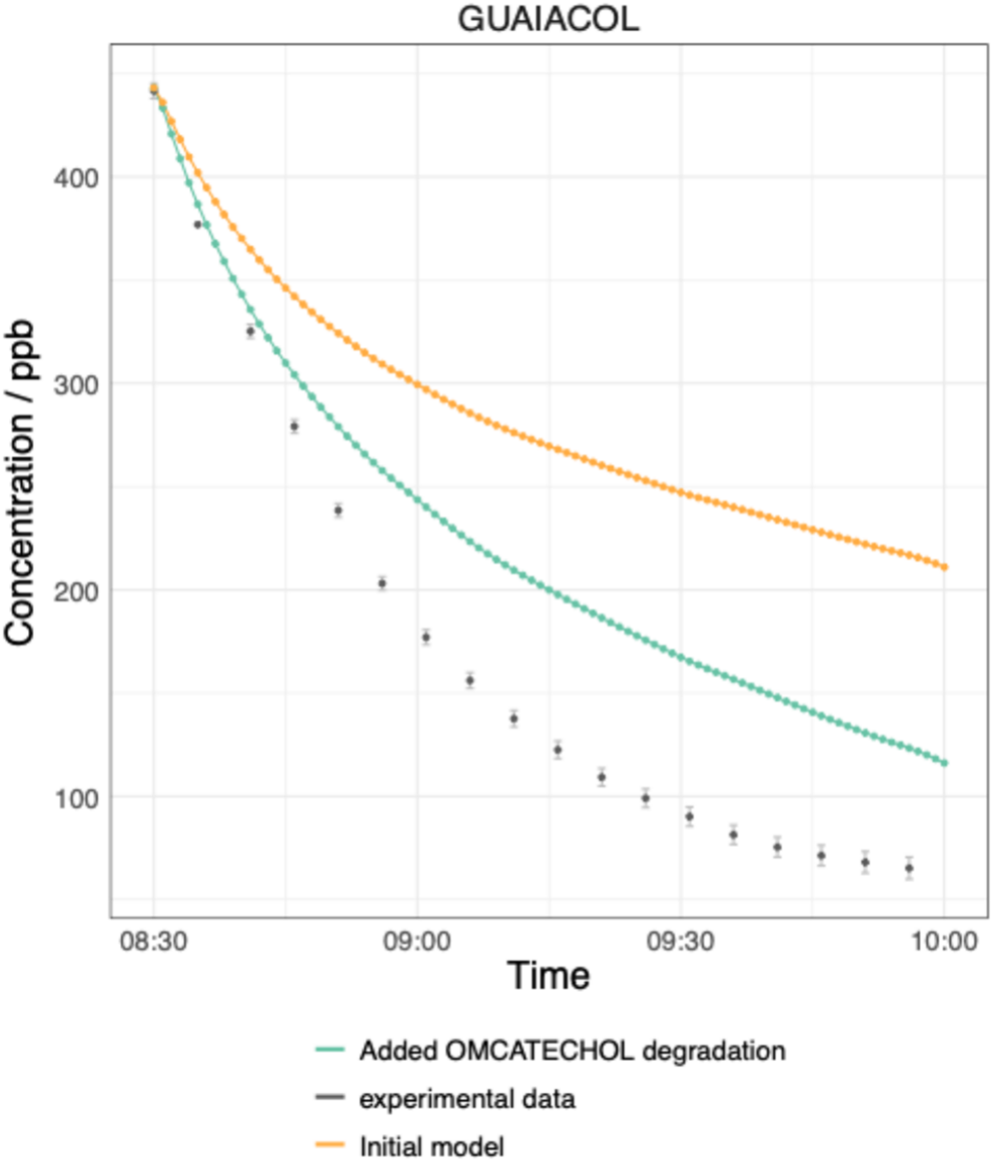
Time series of the guaiacol concentration in parts per billion (ppb) measured by FTIR (gray points) compared to the model concentrations in the initial guaiacol scheme (orange) and after including the degradation of OMCATECHOL RO_2_ (blue). Both model simulations use the SAR-derived OH + guaiacol rate coefficient that is determined in Section 2.1.1.

As the experiments were carried out using HONO photolysis as the OH radical precursor, it is expected that OH will be the major oxidant in this study and hence the dominant reactant with guaiacol. Figure 2 shows the total OH production rate as a function of the top 10 OH production reactions before and after addition of the OMCATECHOL RO_2_ degradation scheme (Scheme S2), which leads to multiple ring opening products. From Figure 2, the addition of the OMCATECHOL chemistry increased the overall OH production rate, primarily from the reaction of HO_2_ with NO, resulting in further radical propagation and greater depletion of the guaiacol precursor than in the initial scheme (Figure 1). A similar analysis was conducted on the rates of RO_2_ production and loss to understand the main RO_2_ conversions propagating the chemistry in the model (Figure S4). It was observed that the major route was via OMCATECHOL RO_2_ and its subsequent products such as peracetyl nitrates (PANs) and unsaturated dicarbonyls. This indicates the importance of OMCATECHOL oxidation products for RO_2_ propagation and OH production, which overall may contribute to the increased OH guaiacol loss in the scheme. Furthermore, the high contribution of PANs to RO_2_ production and loss in the guaiacol scheme (Figure S4) could be important for tropospheric O_3_ and oxidant production in the remote atmosphere^80^ as the injection of biomass burning plumes into the free troposphere can enable PANs to be transported over large distances.^81^

**Figure 2:**
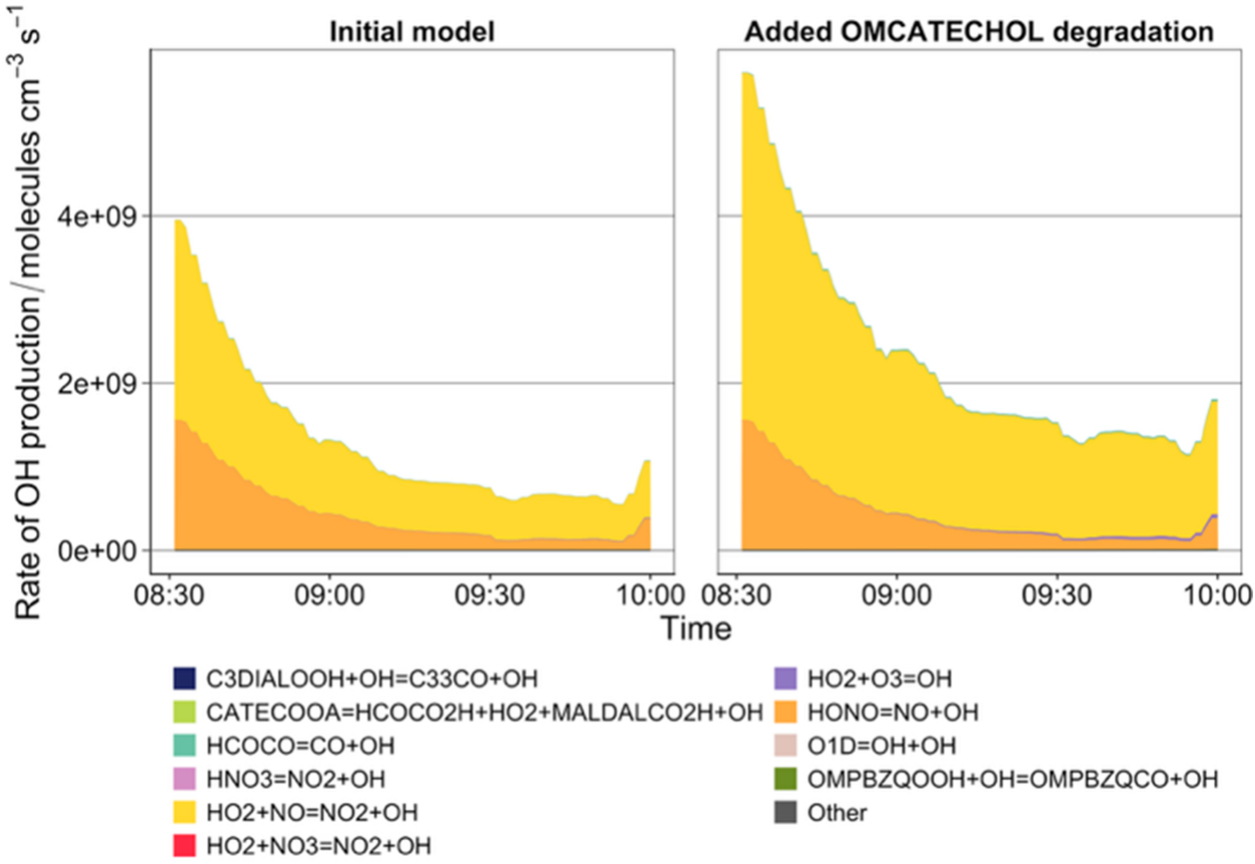
Total OH production rate (molecules cm^–3^ s^–1^) shown as a stack of the top 10 reactions contributing to OH production at 1 min intervals in the initial guaiacol scheme (left) and after adding the degradation of OMCATECHOL RO_2_ (right). Other is defined as the sum of all other reactions outside of the top 10 and each colored ribbon represents the contribution to OH production from individual reactions.

Overall, the initial mechanism shows that further work is required to increase the system reactivity in order to better constrain the guaiacol losses to OH.

#### 3.1.2 Evaluation and Optimization of the Chemical Mechanism

As shown in Figure 1, the model incorporating OMCATECHOL degradation still underpredicted the guaiacol OH loss and simultaneously overpredicted the production of O_3_ (Figure 3). This phenomenon has been previously observed in aromatic chamber modeling systems with missing OH reactivity and underpredicted OH concentrations,^58^ on a similar magnitude as this study. Furthermore, there is still further improvement required in the initial model due to a significant overprediction of glyoxal concentrations of approximately 50 ppb in the initial model, significant underprediction of formaldehyde, and overestimation of the NO to NO_2_ conversion rate (Figure 3). As there are few reactions producing formaldehyde directly in the early stages of the scheme (the auxiliary mechanism contains a wall source), the mechanism is optimized using glyoxal as a direct product from the GUAIAO2 pathway (Scheme 1). However, it is important to note that other possible glyoxal sources may exist, which have not been accounted for. In order to fit the modeled glyoxal concentrations to the observed glyoxal concentrations, the branching ratios between GUAIAO2 and OMCATECHOL formation pathways were adjusted. These pathways were chosen as the 1,2-hydroxyarene product (i.e., OMCATECHOL in Scheme 1) was reported as the dominant product compared to the nitro product or quinone product.^58^ Figure 3 shows the effect of increasing the OMCATECHOL branching ratio and subsequent degradation of OMCATECHOL with OH in 10% increments while simultaneously decreasing the branching ratio to GUAIAO2 by 10% increments on the simulated concentrations of NO, NO_2_, O_3_, guaiacol, glyoxal, HCHO, and HNO_3_. There is a relatively minimal impact on the guaiacol decay (∼8 ppb reduction) or the formation of HCHO, HNO_3_, and O_3_. However, the fit of the modeled NO_2_ decay with the experimental data improves, especially in the latter stages of the simulation, as the OMCATECHOL degradation pathway increases and the GUAIAO2 pathway decreases. However, in all models, the NO to NO_2_ conversion occurs too quickly in the early stages of the experiment, which contributes to the rapid overprediction of O_3_. Constraining to NO and NO_2_ concentrations resulted in an underprediction of O_3_, and the overall reactivity (i.e., OH guaiacol loss) still remained lower than observed, as also seen in the constrained PyCHAM simulation (Figure S3). This suggests that an alternative mechanism that does not involve NO–NO_2_ conversion may be required to yield OH to further the guaiacol loss and ultimately produce radicals, which could also form O_3_. For example, increasing the branching ratios for the reaction of acyl or complex oxygenated RO_2_ species with HO_2_ yielding ROH + O_3_ and/or RO + OH + O_2_.^43^ For acyl RO_2_, these reactions are already accounted for from the SAR.^43^ However, for more complex RO_2_ within the mechanism such as GUAIAO2, OMCATECO2, and OMPBZQO2, which are also the main RO_2_ species propagating the chemistry (Figure S4), the only product from the reaction with HO_2_ is a hydroperoxide (ROOH). It was found that increasing the branching of the RO_2_ + HO_2_ pathway to form RO + OH + O_2_, which has the potential to increase the system reactivity from the production of OH, had a minimal impact on improving OH guaiacol loss (by ∼20 ppb at the end of the experiment) and increasing OH concentration. The increased branching ratio to form RO + OH + O_2_ also led to further O_3_ overprediction (∼13 ppb at the end of the experiment). Furthermore, owing to the high NO*_x_* conditions in this study, NO and NO_2_ were the major HO_2_ sinks, contributing between 87 and 98% of all HO_2_ reactions, while the contribution of HO_2_ + RO_2_ to HO_2_ loss reached a maximum (5%) at the end of the simulation (Figure S5A). For RO_2_ loss, the dominant pathway was via RO_2_ + NO (97%) at the start of the experiment with increasing importance of RO_2_ + NO_2_ by the end of the experiment (42%). However, the reaction of RO_2_ + NO still contributed to 30% of RO_2_ loss by the end of the experiment, while RO_2_ + HO_2_ and RO_2_ + RO_2_ contributed a maximum of 13% and 14%, respectively, to RO_2_ loss by the end of the simulation (Figure S5B). Another possible route for OH production without NO–NO_2_ conversion is RO_2_ intramolecular conversion followed by autoxidation.^77,82,83^ In this study, I-CIMS measurements showed the presence of a homologous series of nonfragmented products from C_7_H_8_O_3_ up to C_7_H_8_O_12_ (Figure S6), indicating the potential formation of highly oxygenated molecules (HOMs) via autoxidation. Currently, autoxidation is not represented in the existing MCM framework and is an ongoing area of research.^84^ Overall, the underprediction of guaiacol OH loss, overprediction of O_3_ formation, and underestimation of OH production is highly complex and likely results from multiple missing chemical processes.

**Figure 3:**
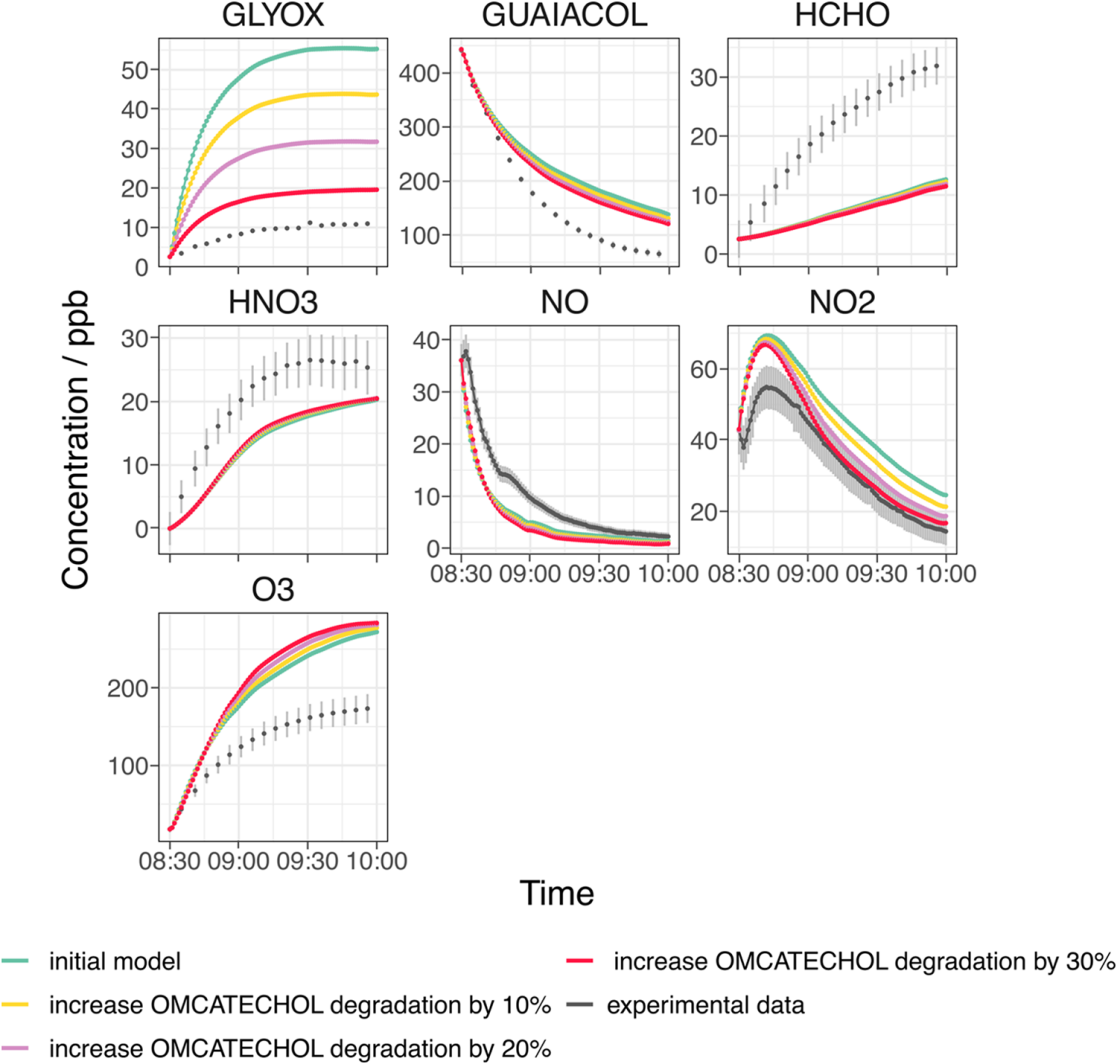
Optimization of the first-generation product branching ratios by increasing the formation of OMCATECHOL and subsequent degradation of OMCATECHOL with OH while simultaneously reducing the formation of the guaiacol RO_2_ (GUAIAO2), assessed by model-measurement comparison of key inorganic and organic markers given in MCM name notation. Models are shown in color and experimental data is in gray. The initial model represents the initial branching ratios calculated by the SAR.

Overall, there is an optimal fit at a 30% increase in OMCATECHOL degradation (Figure 3), which in real terms means the branching ratios of the first-generation products in Scheme 1 are now 17.9% (GUAIAOXMUC), 48.2% (OMCATECHOL), 16% (NGUAIACOL), 6% (CATECHOL), and 11.9% (GUAIAO2). As anticipated, the largest impact of changing the branching ratios is on the prediction of glyoxal, improving by almost 10 ppb for each sequential 10% increase in the OMCATECHOL branching ratio. However, even at the 30% increased branching ratio, there is still an overprediction of the modeled glyoxal concentration by ∼10 ppb. In the current scheme, glyoxal is primarily formed via the degradation of the guaiacol alkoxy radical (GUAIACO) to form a methoxy quinone (OMPBZQONE) or an unsaturated dicarbonyl (OMC5CO14OH) and glyoxal (Scheme 1). The current branching ratio, taken from the MCM toluene mechanism,^58^ favors the unsaturated dicarbonyl product compared to quinone. Therefore, the ratio was adjusted from the original 30:70 (OMPBZQONE:OMC5CO14OH) ratio to 70:30 (Figure S7) to fit the experimental data. Changing this branching ratio did not affect the concentrations of other marker species used in the mechanism evaluation; therefore, using the final fit of 70:30 to favor the quinone product and the 30% increased formation of OMCATECHOL, the mechanism was considered the final optimized version.

#### 3.1.3 Identification of Guaiacol Oxidation Products

Figures 4 and 5 show the time series of the first- and second-generation oxidation products identified using mass spectrometric measurements during the photooxidation experiment with the modeled concentration for each compound. As there are no authentic standards available for every oxidation product to be quantified, the profile is compared between the modeled concentrations in ppb on the left axis (shown in gray) and the species response measured by I-CIMS and PTR-MS (normalized counts per second, ncps) on the right axis (shown in color). Despite the lack of quantification in this study, the comparison of the temporal profiles provides a good indication as to whether the model is capturing the correct temporal chemical evolution during the experiment. It should be noted that previous studies have found time-resolved measurements of semivolatile and highly oxygenated compounds can be influenced by sampling delays associated with gas–wall partitioning in inlet lines.^85,86^ For many of the oxidation products investigated here, authentic standards were not available; however, targeted inlet tests performed under stable chamber conditions indicated rapid signal stabilization and minimal delays for representative VOCs under the measurement conditions applied. While such effects may contribute to minor temporal offsets, the comparison here is based on relative temporal evolution using minute-averaged data, and the overall chemical trends remain robust.

**Figure 4:**
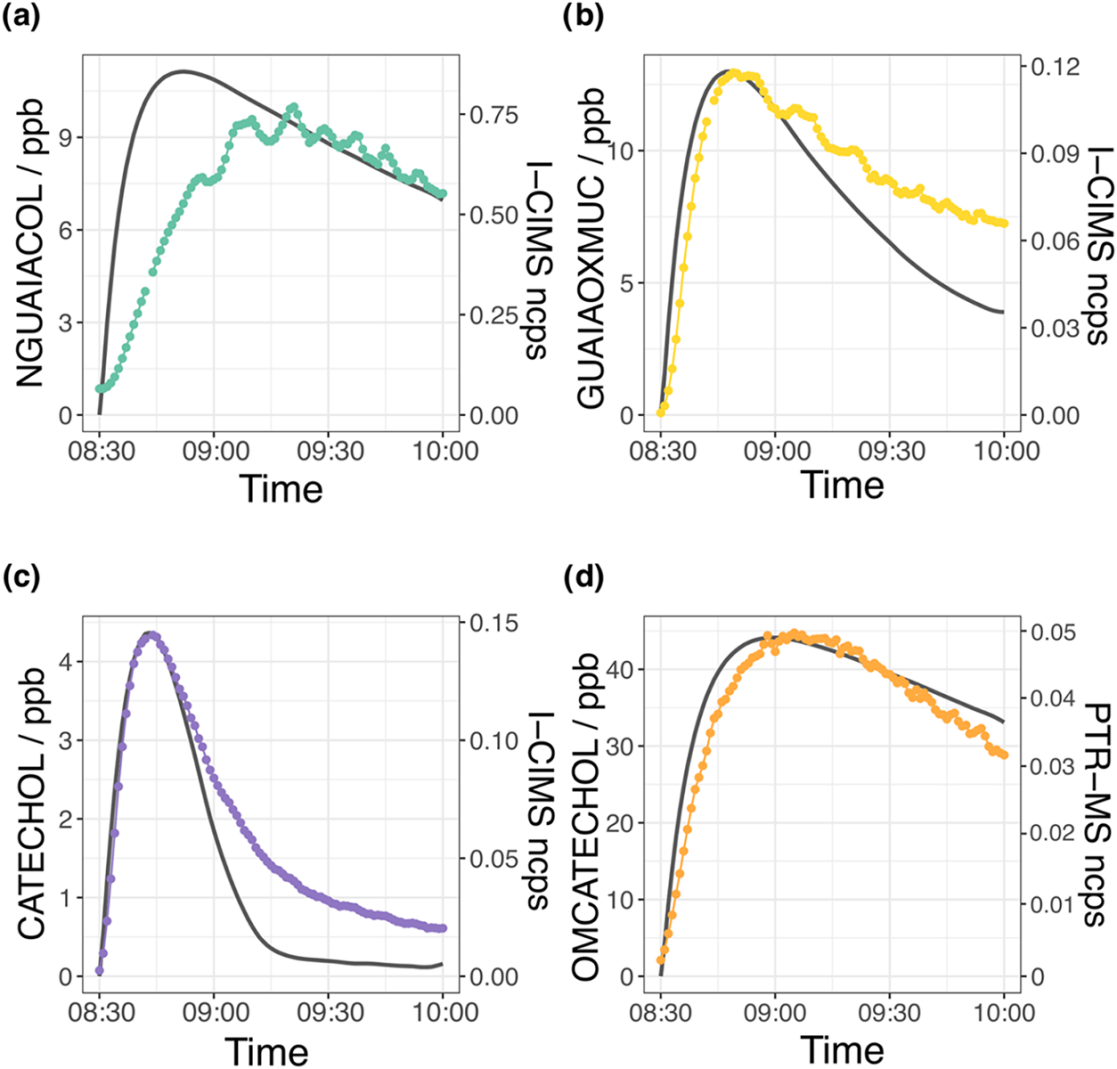
Measured profiles of the guaiacol first-generation products shown in color and normalized counts per second (ncps) on the right axis compared to the modeled concentrations in ppb (gray) on the left axis for (a) nitroguaiacol (NGUAIACOL, *m*/*z* = 296), (b) guaiacol epoxide (GUAIAOXMUC, *m*/*z* = 299), (c) catechol (CATECHOL, *m*/*z* = 237), and (d) methoxycatechol (OMCATECHOL, *m*/*z* = 141).

**Figure 5:**
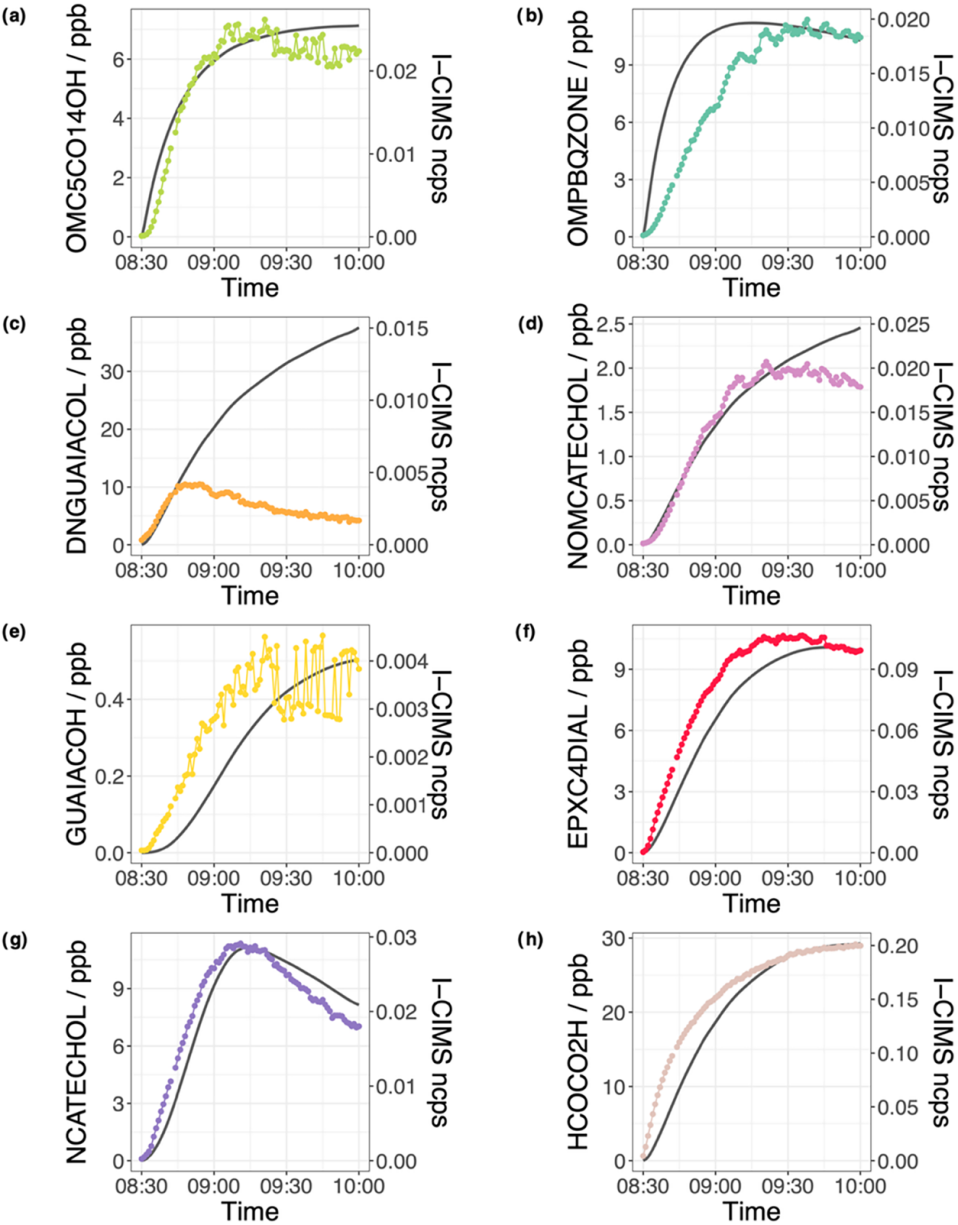
Measured profiles of the guaiacol second-generation products shown in color and normalized counts per second (ncps) on the right axis compared to the modeled concentrations in ppb (gray) on the left axis for (a) unsaturated dicarbonyl (OMC5CO14OH, *m*/*z* = 257), (b) guaiacol quinone (OMPBZQONE, *m*/*z* = 265), (c) dinitroguaiacol (DNGUAIACOL, *m*/*z* = 341), (d) nitromethoxycatechol (NOMCATECHOL, *m*/*z* = 312), (e) bicyclic alcohol (GUAIACOH, *m*/*z* = 317), (f) 2,3-epoxysuccinaldehyde (EPXC4DIAL, *m*/*z* = 227), (g) nitrocatechol (NCATECHOL, *m*/*z* = 282), and (h) glyoxylic acid (HCOCO2H, *m*/*z* = 201).

Generally, the measured profiles of the first-generation products (Scheme 1) show good agreement with the modeled concentration profiles in Figure 4; however, there are some deviations between the modeled and measured profiles. First, the initial production of nitroguaiacol (NGUAIACOL) in the model is too fast compared to that observed, but after approximately 1 h, the decay rate of the two profiles converges to a better agreement. Second, after the first 30 min, there is a deviation in the comparison between modeled guaiacol epoxide (GUAIAOXMUC) concentrations and the I-CIMS signal for the detected C_7_H_8_O_5_I^–^ ion. As the I-CIMS cannot differentiate between isomeric species, there could be other oxidation products forming at the same mass, for instance, the fourth-generation OH addition OMBENZ1345OH product. However, the sum of all known modeled products at *m*/*z* 172 produces a similar profile to GUAIAOXMUC, indicating there are likely to be other missing oxidation products present (Figure S8). For example, C_7_H_8_O_5_ could form via the HOMs autoxidation pathway (Figure S6) or via chemistry analogous to that observed in 1,3,5-trimethylbenzene photooxidation resulting in a bicyclic ketone product.^87^ However, further investigation of these pathways is required.

For second-generation products, there was again generally good agreement between modeled concentration profiles and I-CIMS measurements (Figure 5). However, the model showed some discrepancy when compared to the measurement for nitroaromatics such as dinitroguaiacol (DNGUAIACOL) and methoxynitrocatechol (NOMCATECHOL), although for nitrocatechol (NCATECHOL) the model and measurement profiles were in agreement. In contrast to NGUAIACOL, the model measurement is in agreement initially and then deviates later in the experiment. The timing of the deviation appears to be a function of the number of nitro group functionalities, with a faster rate of deviation for the dinitroaromatic (DNGUAIACOL) compared to the mononitroaromatic (NOMCATECHOL). This is potentially due to their loss to the condensed phase and their differences in volatility, as discussed in the next section.

### 3.2 Particle-Phase Production of Nitroaromatics

As observed in Section 3.1.3, greater differences between the simulated concentrations of guaiacol oxidation products and mass spectrometric measurements appeared for nitroaromatic products. Nitroaromatics are typically low-volatility compounds previously observed in SOA,^37,88,89^ and during the chamber experiment, there is a significant increase in the particle mass concentration from SOA production in the early stages (Figure S1). To understand potential SOA precursor components in the guaiacol scheme, the experiment was modeled using PyCHAM in order to simulate gas-to-particle phase partitioning. The model observed that the addition of bimolecular reactions of organics with NO to form ELVOCs, which can take part in initial particle nucleation, improved the model-measurement comparison, indicating compounds among other organics promoting particle growth, nitrates or nitroaromatics could also be important SOA components. Numerous nitrated organics could be identified using different analysis techniques. For example, offline UHPLC-HRMS filter analysis of guaiacol SOA showed 4-nitroguaiacol could be identified using an authentic standard (Figure S9) and chromatographic peaks were also observed for other nitroaromatics in the scheme, including DNGUAIACOL and NOMCATECHOL. Furthermore, Figure S10 shows the I-CIMS measurements of two possible nitrates formed as well as plausible nitro-HOMs species that are formed during the experiment.

Due to their importance for SOA and brown carbon (BrC) formation,^31,33–37^ nitroaromatics were selected for further study with PyCHAM modeling. Figure 6 shows the PyCHAM-simulated concentrations of gaseous-phase and particulate-phase nitroaromatics derived from guaiacol as well as the potential losses to the chamber wall. From Figure 6, it can be seen that the dominant particle-phase nitroaromatic product is DNGUAIACOL followed by NOMCATECHOL. Considering Figure S2, where the total simulated particulate mass was approximately 350 μg m^–3^ at the end of the simulated period for the case with best agreement with measurement (Section 2.2.2), DNGUAIACOL and NOMCATECHOL contribute 50% and 2%, respectively, to the total particulate mass concentration by this time. This work shows NGUAIACOL is mainly present in the gas phase, or on the wall (Figure S11), which is in agreement with a previous study observing low yields of particulate-phase nitroguaiacol (<0.1%) compared to the gas phase.^18^ For DNGUAIACOL (Figure 6a) and NOMCATECHOL (Figure 6b), the timing at which the particle-phase concentration is competitive to or indeed surpasses that of the gas-phase concentration occurs at a similar timestamp to when the model and measurements begin to diverge for these compounds (Figure 5). This suggests that the discrepancy between the modeled gas-phase concentrations and I-CIMS measurements is in part explained by gas-to-particle phase partitioning. However, PyCHAM fails to reproduce the observed decrease in gaseous DNGUAIACOL seen in the I-CIMS measurement (Figure 5), which may result from other deficiencies in the scheme. Furthermore, it is important to note that in the ambient atmosphere, the absorbing particle mass may be lower compared with this chamber study, returning lower SOA yields. Nonetheless, these results indicate that the atmosphere nitroaromatics, and in particular dinitroguaiacol, are important in the early stages of guaiacol SOA formation, which could have potentially significant implications for BrC formation and hence air quality and climate from biomass burning in aged plumes.

**Figure 6:**
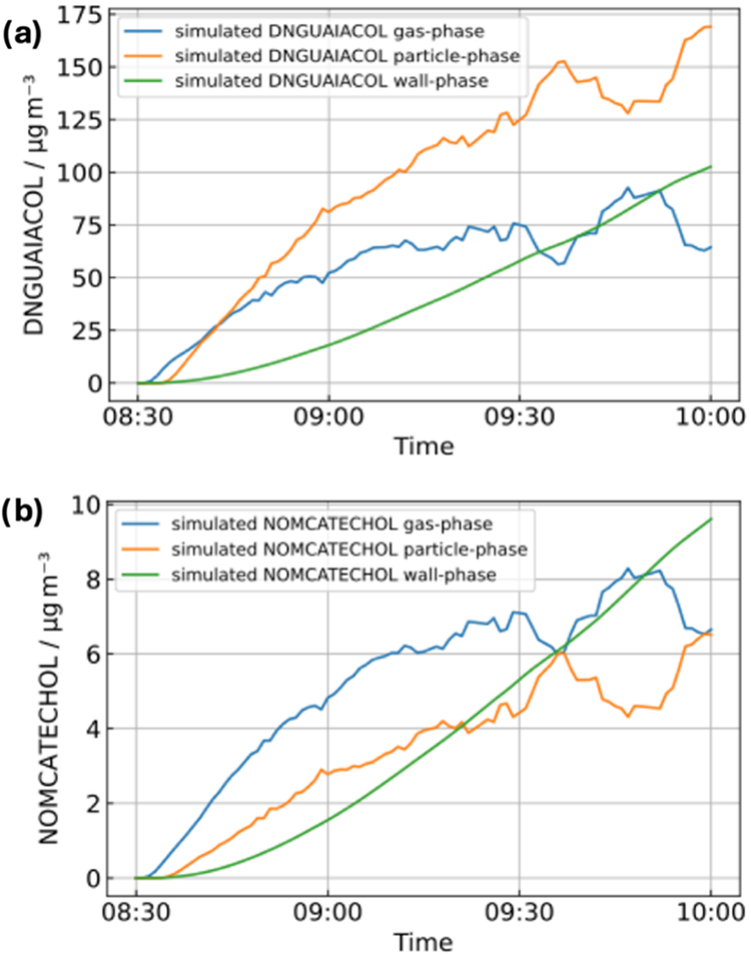
Simulated mass concentrations in μg m^–3^ of gaseous, particulate, and wall-phase guaiacol nitroaromatics: (a) dinitroguaiacol (DNGUAIACOL) and (b) nitromethoxycatechol (NOMCATECHOL).

## 4 Conclusions

A new near-explicit gas-phase photooxidation mechanism of guaiacol was developed for inclusion into the Master Chemical Mechanism framework, evaluated using chamber box model simulations, and optimized against a photooxidation experiment conducted at the EUPHORE chamber. Mechanism evaluation and optimization was assessed against key inorganic and organic oxidation markers and the precursor VOC loss. During mechanism optimization, it was found that incorporating the OH oxidation of methoxycatechol (OMCATECHOL), resulted in greater radical formation from ring-opened products and improved the modeled guaiacol OH loss. However, after optimization, the mechanism still underpredicted guaiacol OH loss and overpredicted O_3_ production, which is a phenomenon previously observed in other aromatic systems in chamber studies with missing OH reactivity.^58^ Additionally, the modeled rate of NO to NO_2_ conversion occurred too quickly, which contributed to the rapid overprediction of the O_3_ concentration. These findings indicate that RO_2_ chemistry propagating OH production without NO to NO_2_ conversion could improve the model-measurement comparison. One such possibility is via a unimolecular autoxidation process, but there are other possible chemical pathways that require further investigation. To improve mechanism evaluation in this work, future studies are needed to investigate autoxidation and other potentially important low NO_*x*_ pathways that can propagate RO_2_ chemistry without NO–NO_2_ conversion during aromatic BBVOC oxidation, to enable the development of new SAR parametrizations. However, despite the underprediction of guaiacol OH loss, the model was able to reasonably reproduce the temporal evolution of first- and second-generation guaiacol oxidation products. Coupling the optimized gas-phase scheme with a chamber microphysics particle box model, PyCHAM, showed that the differences seen in the model and measured temporal profiles for the nitroaromatic products could be attributed to gas-to-particle phase partitioning, which is supported by offline SOA filter composition analysis using HPLC-ESI(−)-HRMS. This observation has potentially important implications for BrC and SOA formation in aged biomass burning plumes. Finally, it is recommended that experimental chamber studies use major oxidation products as the precursor compounds; for example, methoxycatechol would enable a greater understanding of OH production in this study. Overall, this work provides a new gas-phase mechanism and detailed experimental evaluation of an important and relatively understudied methoxyphenol emitted from biomass burning to better inform chemical modeling assessments of the impact of biomass burning on air quality and climate, which is especially important in the current global trend of increasing wildfire severity and frequency.

## Supplementary Material



## Data Availability

The experimental data from the EUPHORE chamber experiment is openly available at https://data.eurochamp.org/data-access/chamber-experiments/#/datasets/6d36650b-299e-4d94-8a8c-304e8a897edc (10.25326/B5Q7-MX18). The MCM optimized guaiacol chamber mechanism file in facsimile format, suitable use in various model applications (e.g., AtChem2 ^67^), is available at 10.15124/a542aac8-407b-45b6-a001-946fc0c025c9. The PyCHAM simulation code can be accessed at https://github.com/simonom/PyCHAM/releases/tag/v5.6.0.

## Notes

Chemical schemes of the guaiacol mechanism showing initial branching ratios to first-generation guaiacol products and additional chemistry of the OMCATECHOL RO_2_; Supporting figures of particle number concentrations, I-CIMS measurements of guaiacol oxidation products, PyCHAM simulation optimization and results, RO_2_ and HO_2_ production/loss in the model simulations and offline LC-MS analysis of guaiacol-derived nitroaromatics; and Supporting text describing parameters for the offline chemical compositional LC-MS analysis of guaiacol-derived SOA and further parametrizations surrounding the optimization of particle growth in the PyCHAM simulation (PDF)

## ∇

Department of Environmental Toxicology, University of California Davis, Davis, California 95616, United States
