# Supplementary material for: Mechanism Development and Evaluation of the Gas-Phase Photooxidation of Guaiacol: Insights from a Chamber Study
Source: ACS EST Air. 2026 Mar 16;3(4):1018–31. doi: 10.1021/acsestair.5c00420 (PMC13077636; doi:10.1021/acsestair.5c00420)
Supplement: Supplementary file 1 [file ea5c00420_si_001.pdf]

Supporting Information

Mechanism development and evaluation of the gas-phase photo-oxidation of guaiacol: Insights from a chamber study

Author(s): Evans*, Rhianna; Soler, Ruben; Vera, Teresa; Ródenas, Milagros; Borras, Esther; Gomez, Tatiana; Bryant, Daniel; Mayhew, Alfred; Shaw, David; O'Meara, Simon Patrick; Munoz, Amalia; Hamilton, Jacqueline; Rickard*, Andrew

*Correspondence to rhievans@ucdavis.edu and andrew.rickard@york.ac.uk

Table of Contents

Supporting Figures and Schemes 2

Scheme S1: Branching ratios to 1st generation guaiacol products 2
Scheme S2: Additional chemistry of the OMCATECHOL RO_2 3
Figure S1: Particle number concentration timeseries from SMPS measurement 4
Figure S2: PyCHAM simulated particle mass concentration for varying parameters of gas-wall partitioning C_w and k_e coefficients. 4
Figure S3: PyCHAM simulated guaiacol gas phase concentration 5
Figure S4 – Top 10 reactions contributing to the total rate of RO_2 production and RO_2 loss in the model 6
Figure S5 – Contribution of the major grouped reactions (ie. all RO2 are summed) to A) HO2 loss and B) RO2 loss in the model 7
Figure S6 – I-CIMS measurements of the potential HOMs homologous series from guaiacol 8
Figure S7 - Optimisation of the quinone-to-unsaturated dicarbonyl branching ratio 9
Figure S8 - Sum of all modelled species with *m/z* 172 and formula C7H8O5 10
Figure S9 - UHPLC-HRMS extracted chromatograms and corresponding ESI(-) mass spectra of nitroaromatics formed from guaiacol oxidation 11
Figure S10 - I-CIMS measurements of potential nitrate products and guaiacol nitro functionalised HOMs products 12
Figure S11- Simulated mass concentrations of gaseous, particulate and wall-phase guaiacol nitroguaiacol using PyCHAM 13

Supplementary Text 13

Ultra-High-Performance Liquid Chromatography coupled to High Resolution Mass Spectrometry 13
Parameterisation of ELVOC early stage particle growth in PyCHAM 14-15

Supporting Figures and Schemes

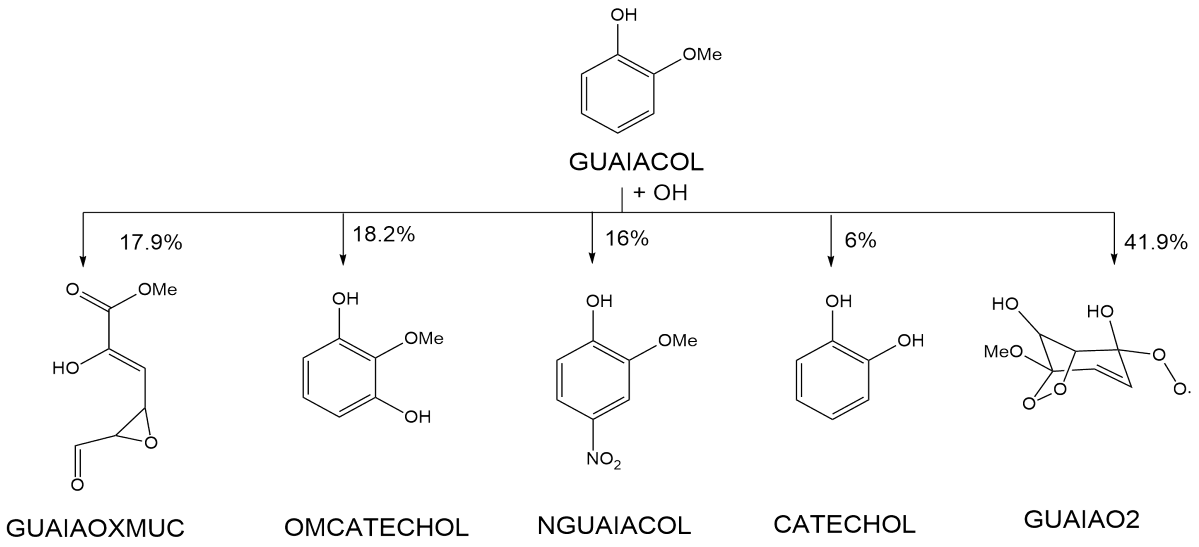

Scheme S1 – Scheme showing 1st generation guaiacol oxidation products and the corresponding branching ratios determined from literature yields and the SAR calculations.

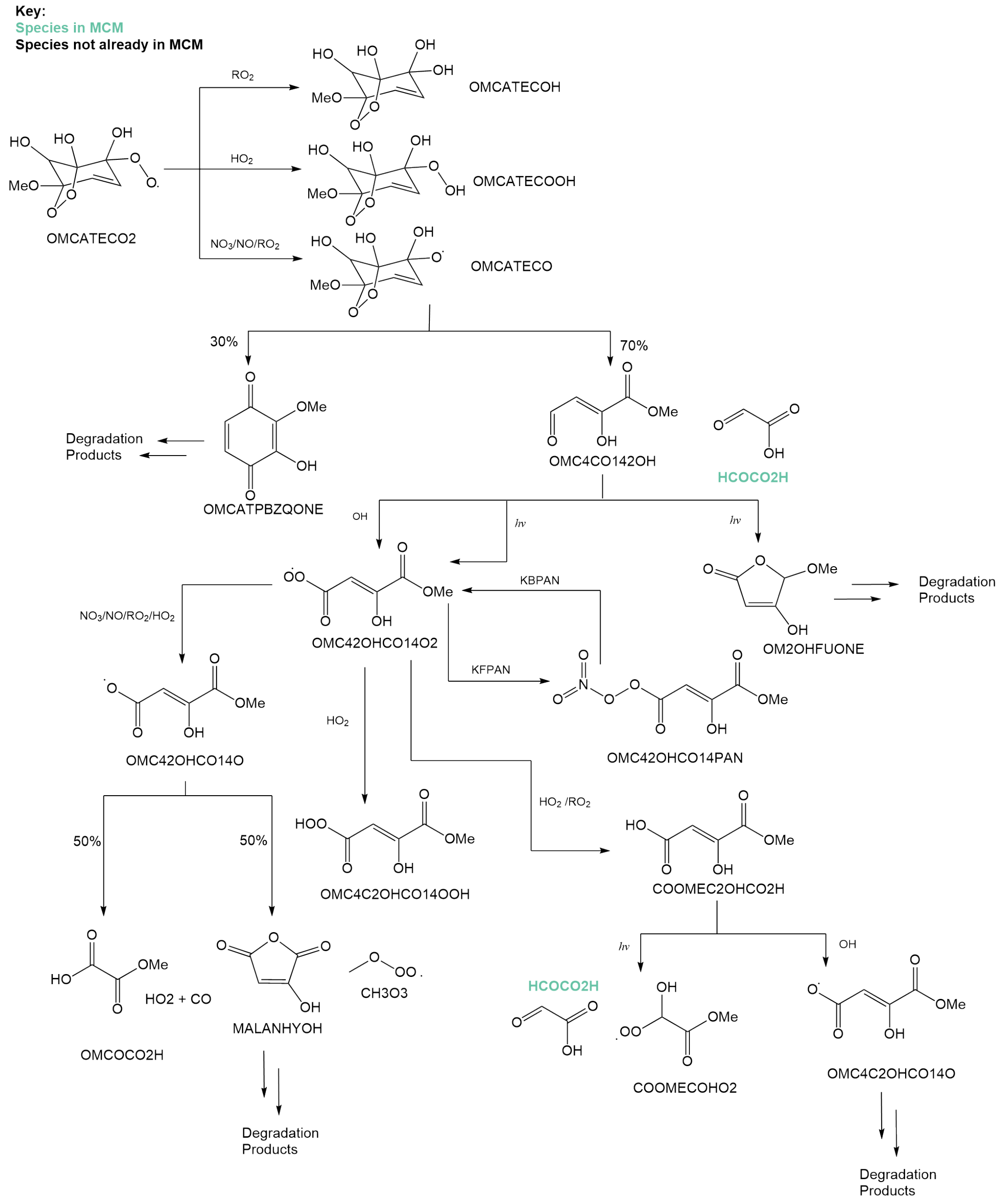

Scheme S2 – Additional chemistry of the RO_2 derived from the OH addition product, OMCATECHOL, included to improve the model evaluation.

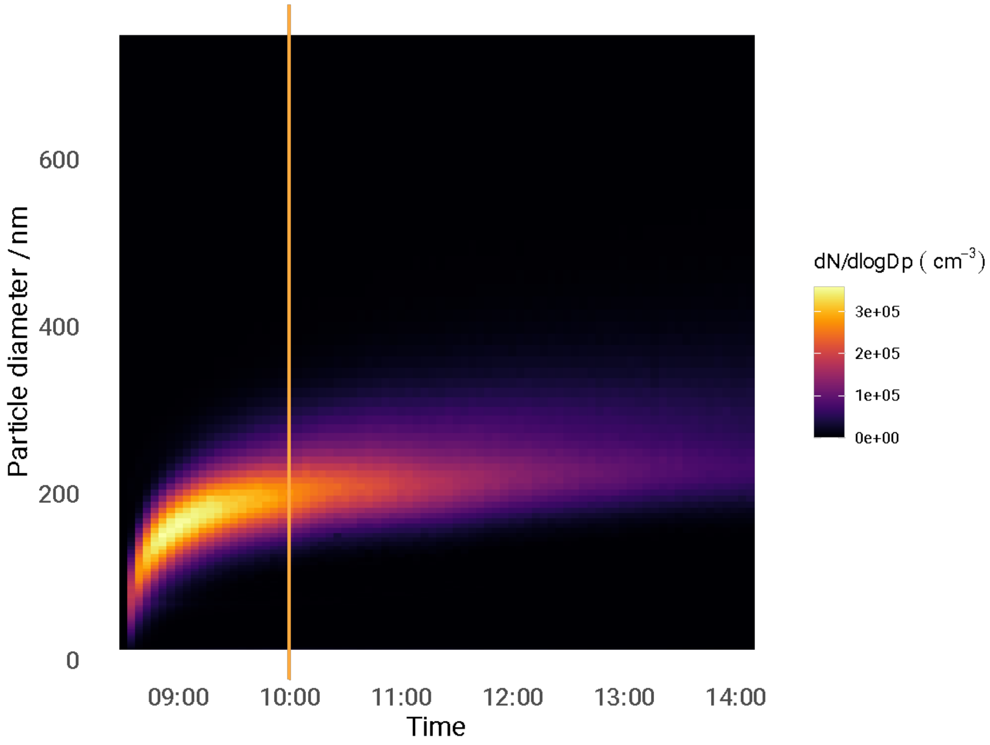

Figure S1 - Particle number concentration measured by SMPS during the guaiacol photo-oxidation experiment. The orange line represents the end of the time period used in the chamber box modelling when the majority of the particle growth occurs.

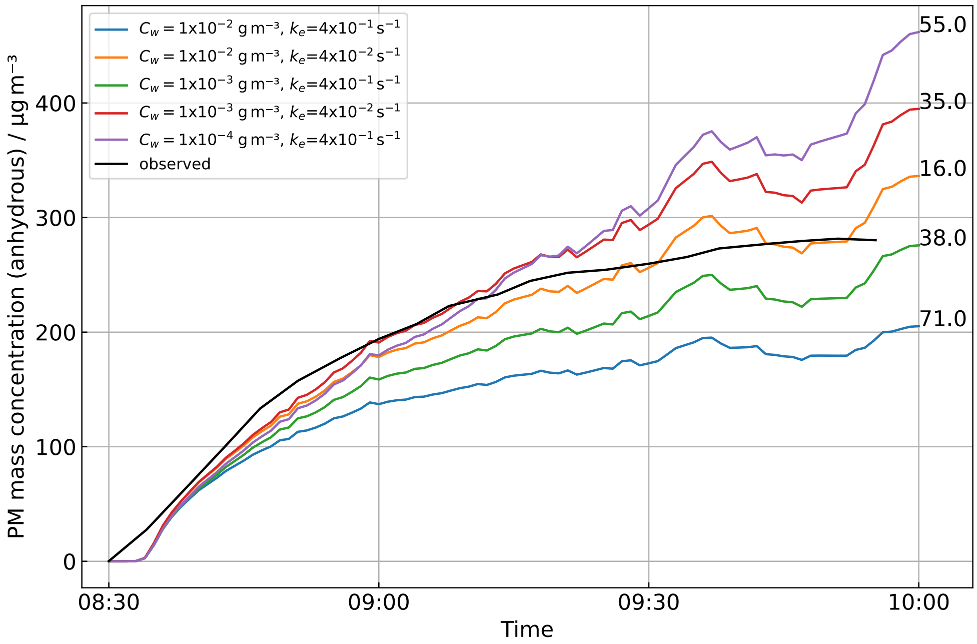

Figure S2 - Simulated particle matter (PM) mass concentration from PyCHAM compared to the observed mass concentration for varying parameters of gas-wall partitioning C_w and k_e coefficients used in the model set up. The root mean squared error (RMSE) between simulated and observed particle mass concentration is shown to the right of each trace.

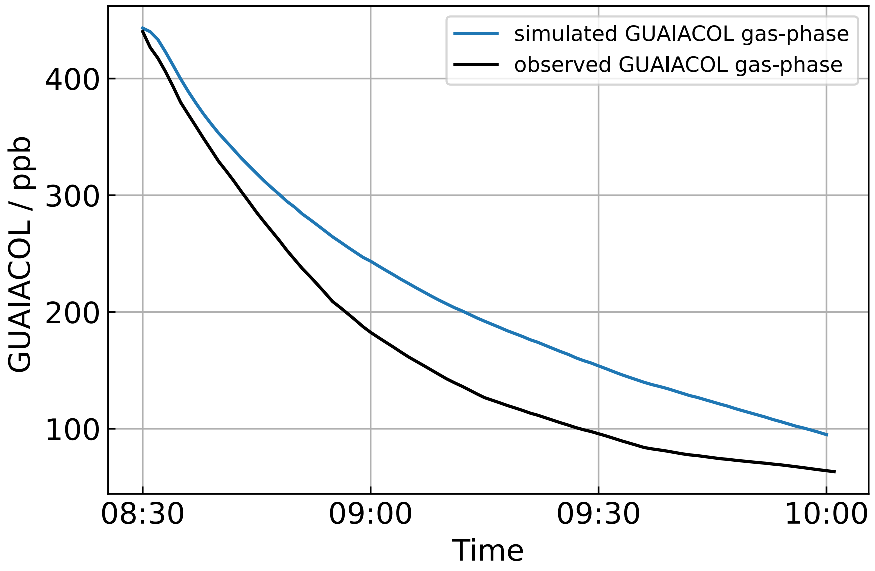

Figure S3 – PyCHAM Simulated guaiacol gas phase concentration when constrained to HONO, NO, NO_2, O_3 and HCHO compared against observed for the chosen gas-wall partitioning parameters (1×10^{-3} g m^{-3} for C_w and 4×10^{-1} s^{-1} for k_w).

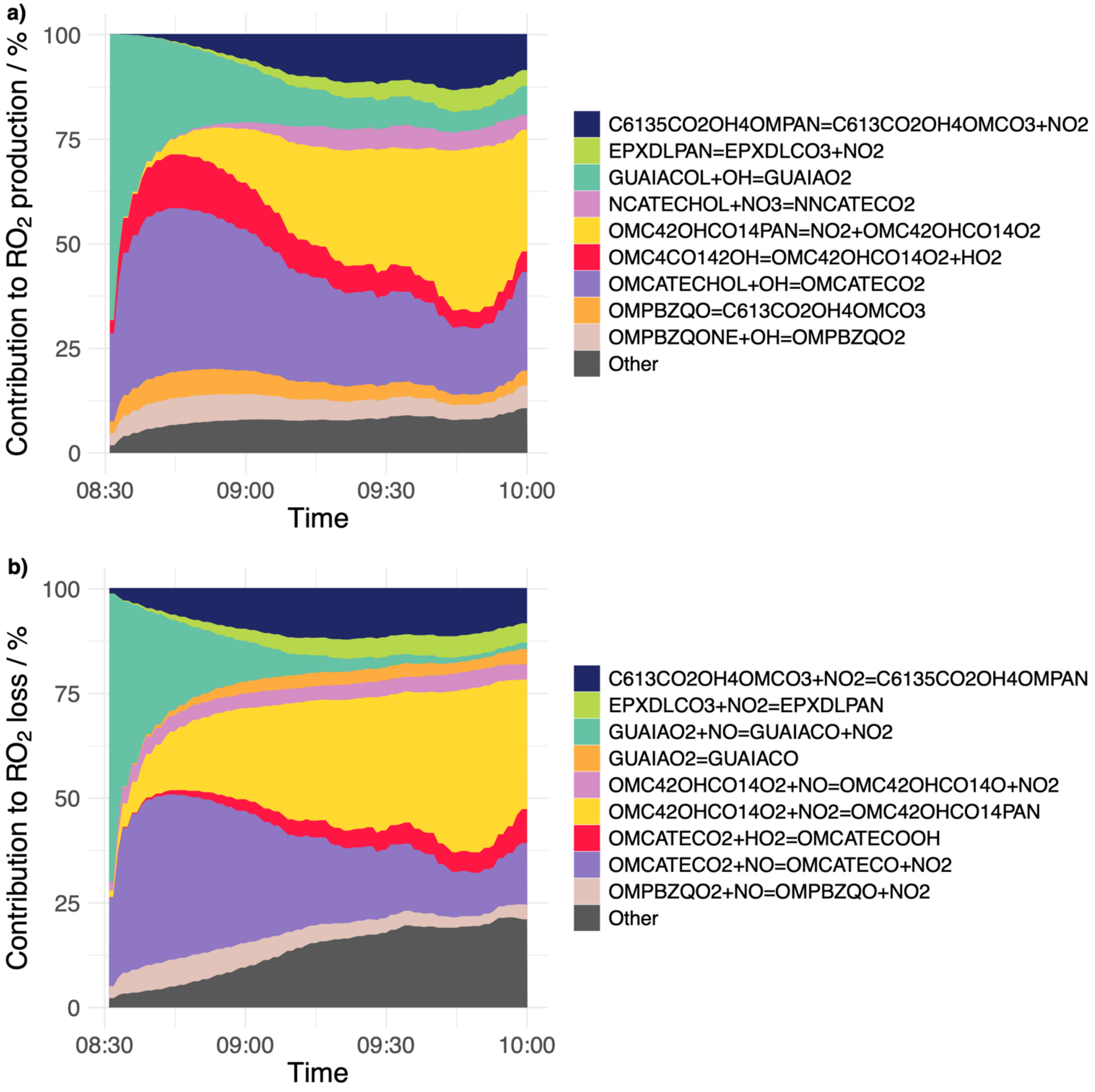

Figure S4 – Stacked depiction of the top 10 reactions contributing to the total rate of RO_2 production and RO_2 loss at 1 minute intervals throughout the model duration, shown as a percentage contribution (%). Each ribbon represents the percentage contribution of an individual reaction. Other is defined as the sum of all reactions outside the top 9.

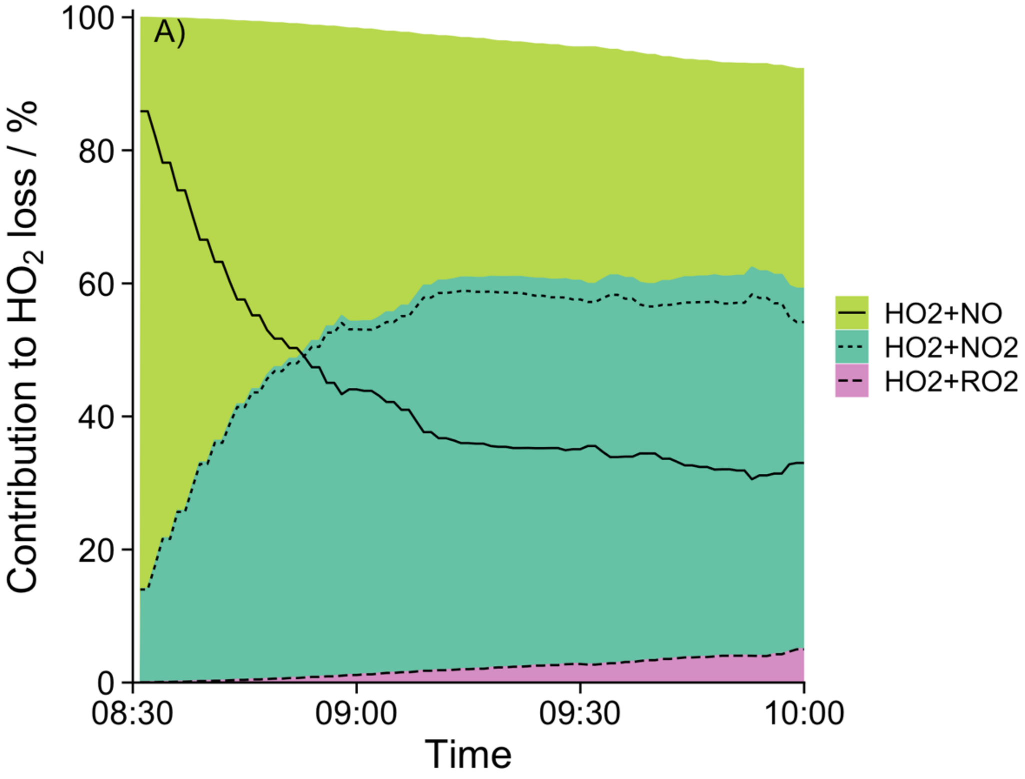

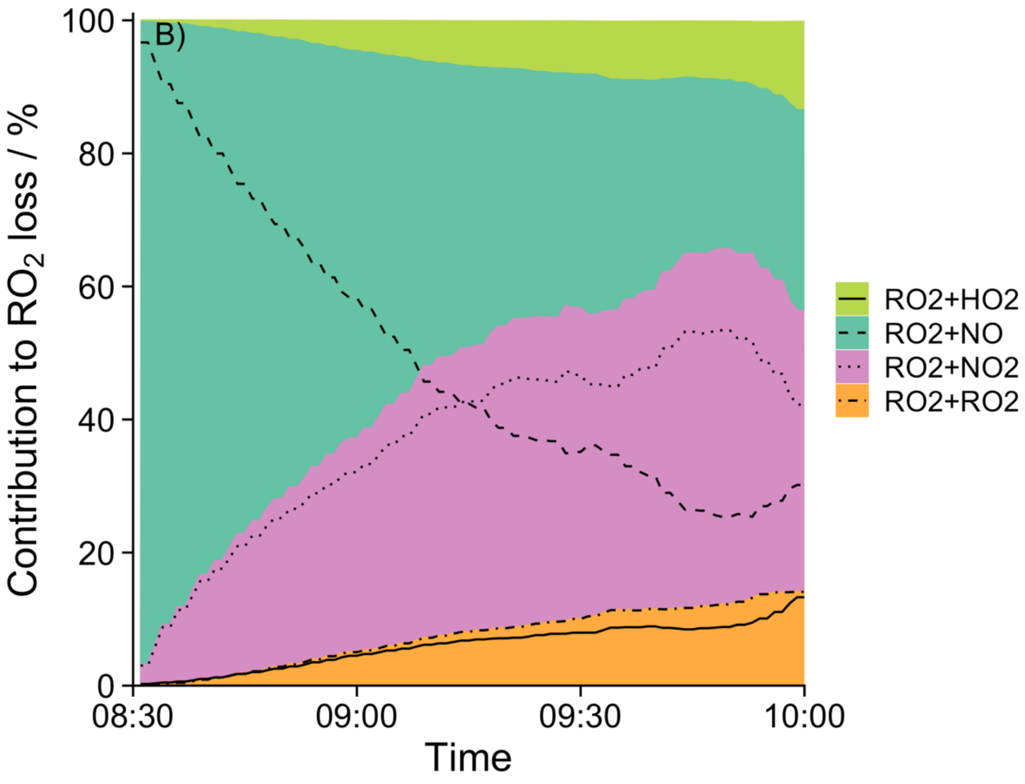

Figure S5: Contribution of the major grouped reactions (ie. all RO_2 are summed) to A) HO_2 loss and B) RO_2 loss. at 1 minute intervals throughout the model duration, shown as a percentage contribution (%). Each ribbon represents the percentage contribution of an individual reaction which are then stacked. The black line tracers show the exact contribution of each reaction over time.

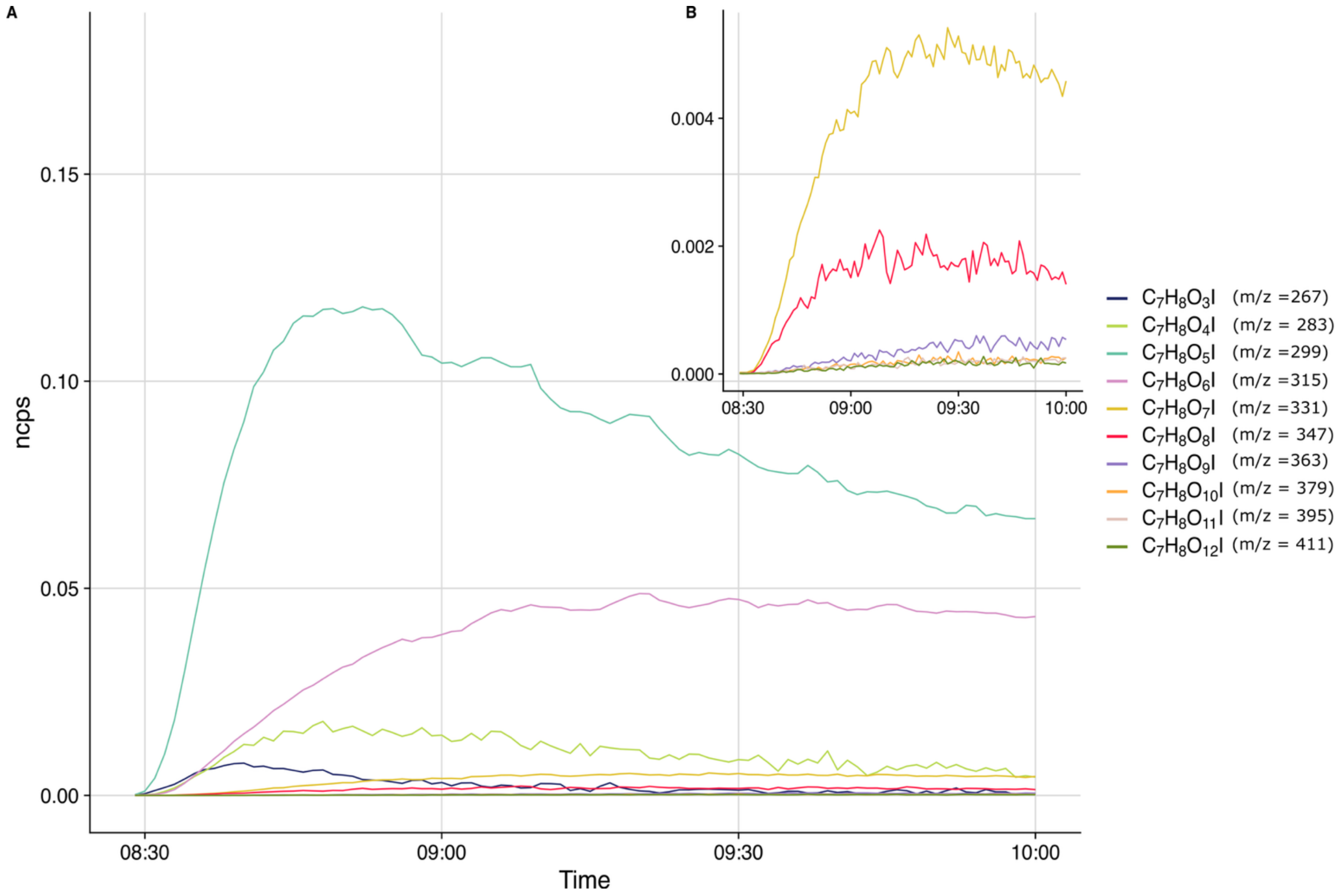

Figure S6 - I-CIMS measurements of the potential HOMs homologous series from $C_7H_8O_3$ to $C_7H_8O_{12}$ deriving from guaiacol ($C_7H_8O_2$). Panel B shows an inset plot for the later generational and increasingly oxygenated HOMs.

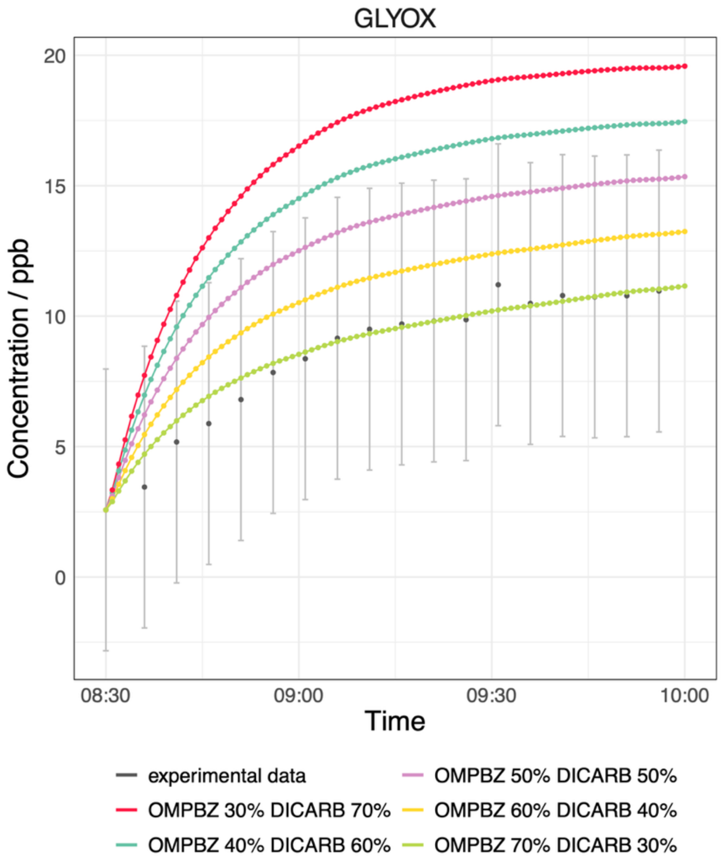

Figure S7 - Optimisation of the quinone-to-unsaturated dicarbonyl branching ratio (OMPBZ:DICARB) from the initial value of 30:70 (taken from toluene[56,59]) to 70:30, assessed via FTIR measurements of the glyoxal marker formed on the unsaturated dicarbonyl branch.

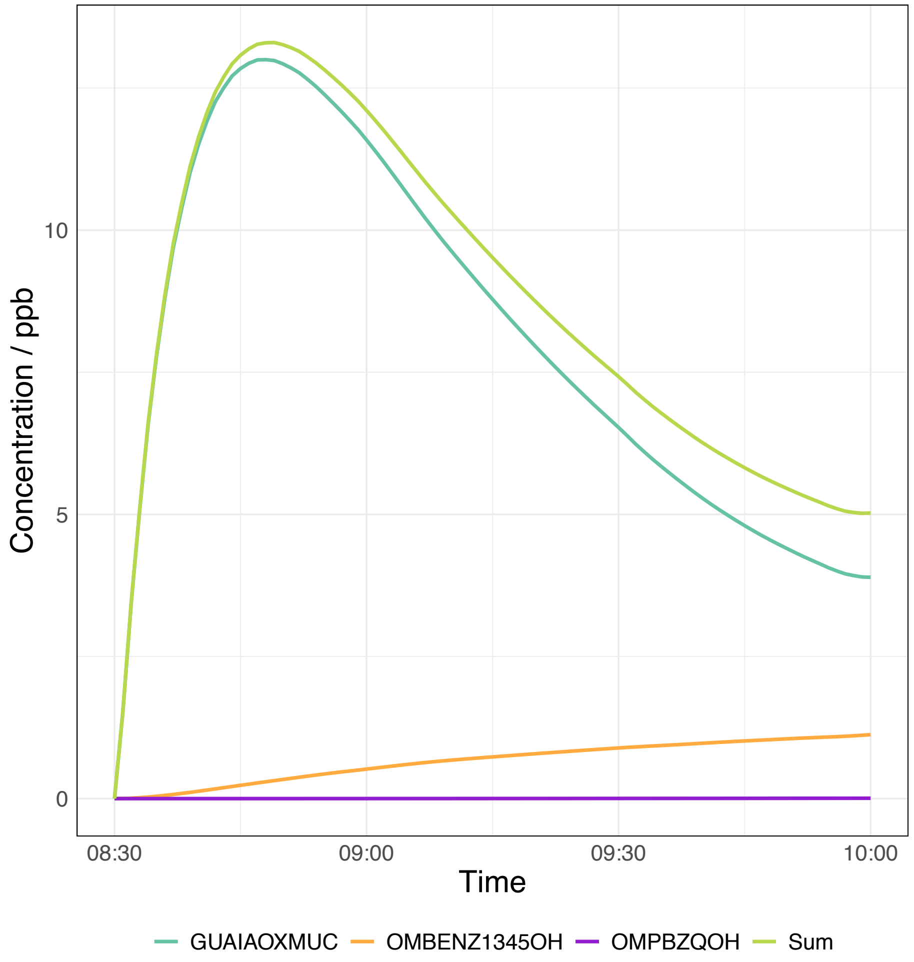

Figure S8 – Sum of all modelled species with m/z 172 and formula $C_7H_8O_5$ within the current guaiacol sceheme and the individual profiles of each compound.

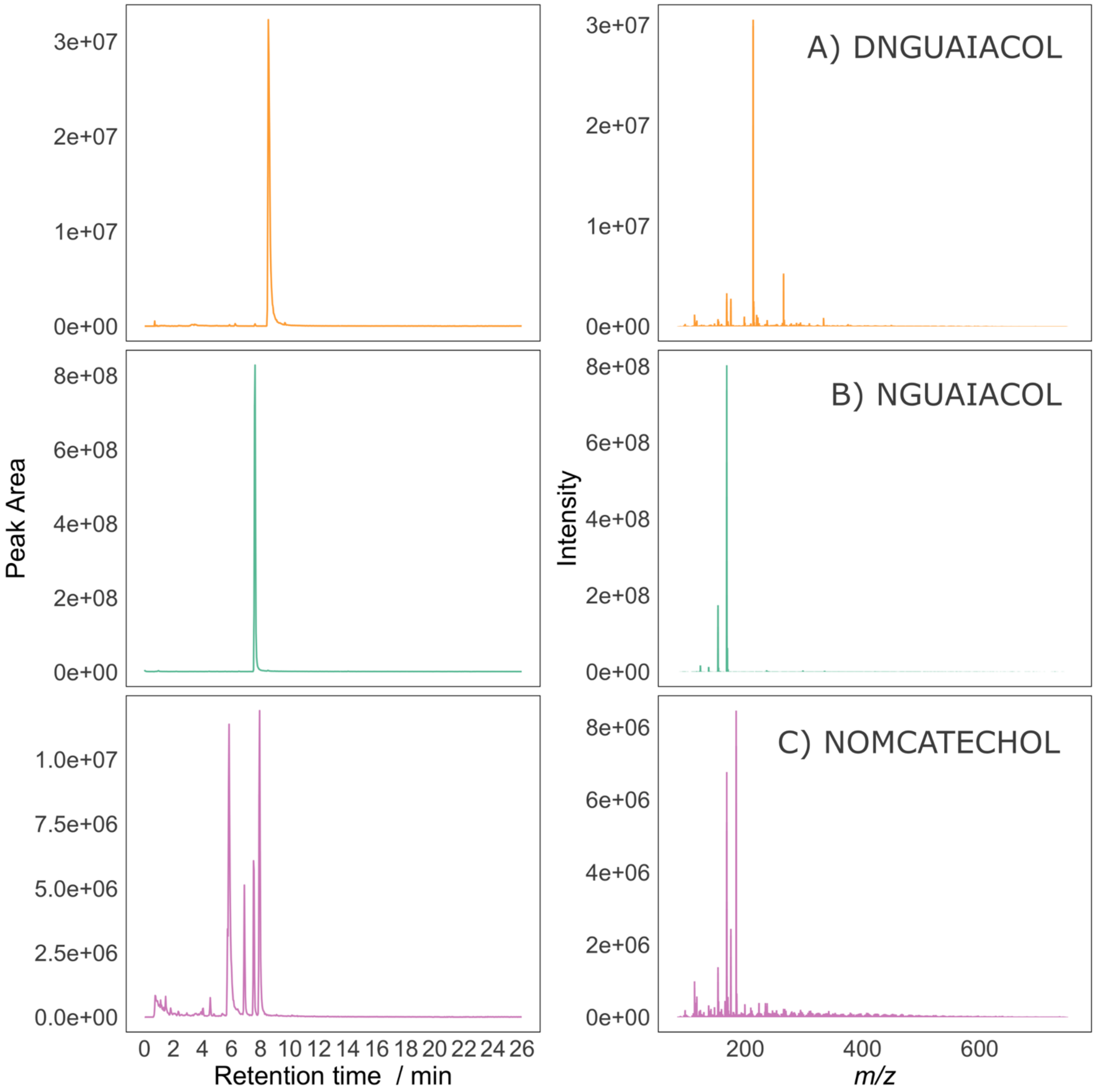

Figure S9 - UHPLC-HRMS extracted ion chromatogram (left panel) and corresponding ESI(-) mass spectra (right panel) of A) dinitroguaiacol (DNGUAIACOL, *m/z* = 213.0168), B) nitroguaiacol (NGUAIACOL, *m/z* = 168.0305), and C) nitromethoxycatechol (NOMCATECHOL, *m/z* = 184.0257). 4-nitroguaiacol could be identified as the NGUAIACOL isomer via an authentic standard.

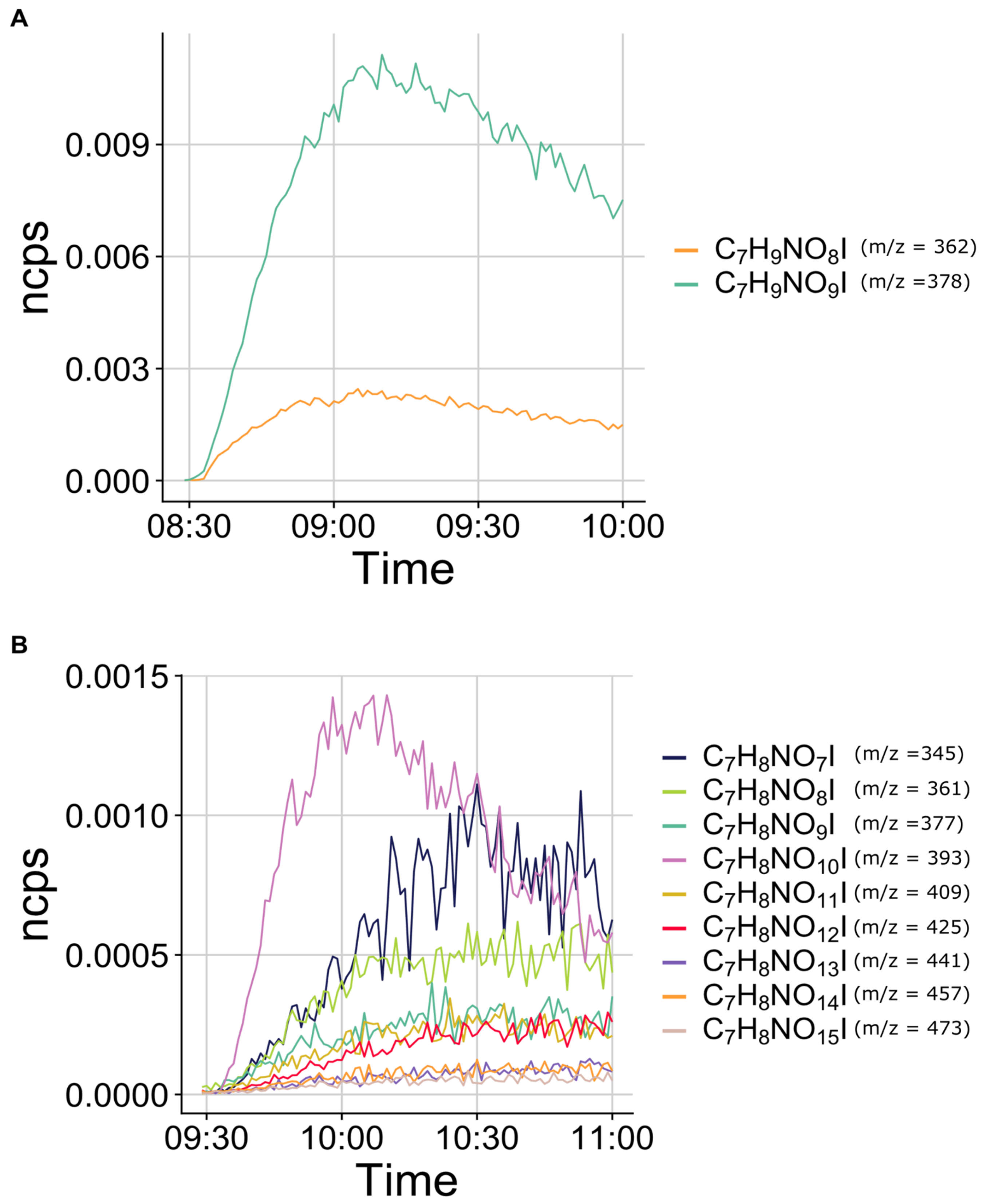

Figure S10 - I-CIMS measurements of potential a) nitrate products with molecular formula $C_7H_9NO_8$ and $C_7H_9NO_9$ derived from the guaiacol RO_2 (GUAIACO2) and from the guaiacol epoxide (GUAIAOXMUC) respectively and b) guaiacol HOMs products identified in Figure S5 with a potential additional nitro group ($-NO_2$) functionality.

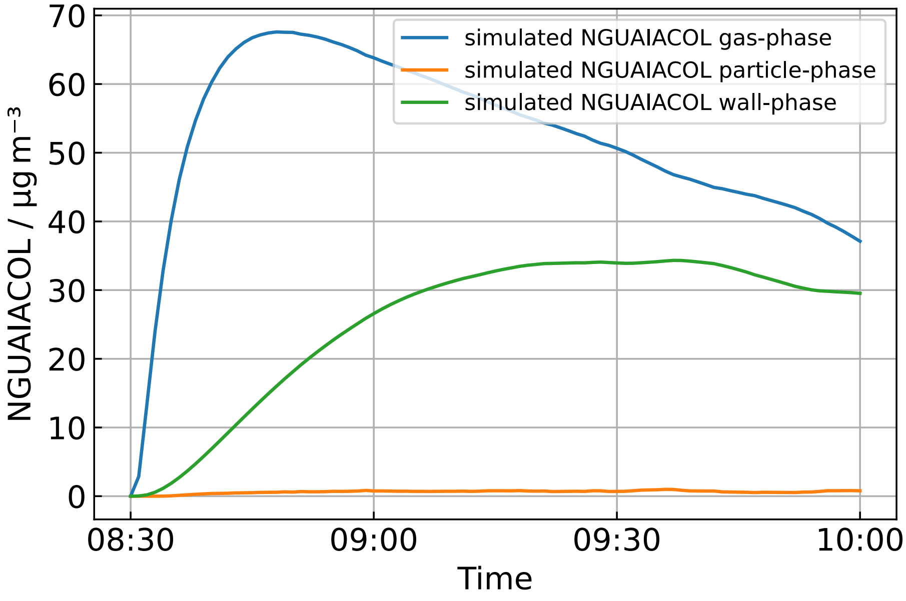

Figure S11 - Simulated mass concentrations in µgm^{-3} of gaseous, particulate and wall-phase guaiacol nitroguaiacol (NGUAIACOL)

Supplementary Text

Ultra-High-Performance Liquid Chromatography coupled to High Resolution Mass Spectrometry

The offline filters were stored in the freezer at -20°C at EUPHORE until the end of the campaign before being transported on ice to the United Kingdom and characterised at the University of York using an Ultimate 3000 UHPLC (Thermo Scientific, USA) coupled to a Q Exactive Orbitrap MS (Thermo Fisher Scientific, USA) with heated electrospray ionisation (HESI). The filters were extracted based on the method used in Bryant et al, 2023. The 47 mm quartz filters were cut into 1 cm^2 pieces, placed in a 20 mL glass vial and 8 mL of methanol (LC-MS Optima Grade) was added. The resulting 8 mL solution was sonicated for 45 minutes, using ice packs to lower the temperature of the water bath. The methanol extract was transferred to a second 20 mL glass vial using a 0.22 μm syringe filter (Millipore) then 2 mL of methanol was used to wash through the syringe filter yielding a 10mL extract. The 10 mL extract was subsequently dried using a Genevac vacuum solvent evaporator. The samples were reconstituted in 200-300 μL 90:10 H_2O (LC-MS Optima Grade): MeOH (LC-MS Optima Grade) for UHPLC-HRMS analysis.

Compound separation was achieved using a reversed phase C_{18} 2.6 µm × 2.1 mm × 100 mm Accucore column held at 40°C. The mobile phase consisted of 0.1 % (*v/v* \%) formic acid (Acros Organics) in water (A, LC-MS Optima Grade) and methanol (B, LC-MS Optima Grade). A gradient elution was used, starting at 90 % (A) with a 1-minute post injection hold, decreasing to 10 % (A) at 26 minutes before returning to the starting conditions at 28 minutes. A final 2-minute hold at 10 % (A) allowed to the column to re-equilibrate. The flow rate was set to 0.3 mL min^{-1} and prior to analysis samples were stored in an autosampler tray at 4°C. The injection volume was set to 4 µL. The HESI was operated under the following conditions: a spray voltage of 4 kV, a capillary and auxiliary gas temperature of 320 °C, a sheath gas flow rate of 45 (arb.) and an auxiliary gas flow rate of 10 (arb.) Spectra were acquired in negative mode using $ddMS^2$. The scan range was set to a mass-to-charge ratio (*m/z*) of 85 to 750, with a mass resolution of 140,000. Tandem mass spectrometry was performed using a higher collision dissociation with a stepped normalised collision energy of 10, 20 and 45. In each scan the 10 most abundant species were selected for MS^2 fragmentation. The samples were analysed once with solvent blanks every 10 samples. Spectra were acquired from XCalibur 4.3 (Thermo Scientific, USA).

Parameterisation of ELVOC early stage particle growth in PyCHAM

The generation of an extremely low volatility product (ELVOC) from guaiacol (GUAIACOL) oxidation was represented through the simplified mechanism below, where ELVOCp is a relatively volatile precursor to ELVOC and ELVOCpp is a relatively volatile termination product that acts as an alternative termination product to ELVOC. The branching ratio for GUAIACOL + OH = ELVOCp was tuned to give the observed growth of newly nucleated particles, whilst the rate coefficient for ELVOCp = ELVOCpp was tuned such that it was sufficiently competitive with the ELVOCp + NO = ELVOC to effectively cease production of ELVOC later in the experiment as RO2 concentration increased. Rate coefficients for ELVOCp + NO = ELVOC and ELVOCp + HO2 = ELVOCpp are consistent with others for peroxy radical reaction in the Master Chemical Mechanism:

% 6.99D-11*0.04 : GUAIACOL + OH = ELVOCp ;

% KRO2NO : ELVOCp + NO = ELVOC ;

% KRO2HO2 : ELVOCp + HO2 = ELVOCpp ;

% 4.0D-10*RO2 : ELVOCp = ELVOCpp ;
